# The Use of Customized Three-Dimensionally Printed Mandible Prostheses with a Pressure-Reducing Device: A Finite Element Analysis in Different Chewing Positions, Biomechanical Testing, and In Vivo Animal Study Using Lanyu Pigs

**DOI:** 10.1155/2022/9880454

**Published:** 2022-03-16

**Authors:** Chun-Feng Chen, Chun-Ming Chen, Han-Sheng Chen, Wei-Chin Huang, Yung-Chung Chen, Hung-Chih Chang, Sung-Ho Liu, Tsung-Lung Yang, Ling-Lin Wang, Ping-Ho Chen

**Affiliations:** ^1^School of Dentistry, College of Dental Medicine, Kaohsiung Medical University, Kaohsiung, Taiwan; ^2^Department of Oral and Maxillofacial Surgery, Kaohsiung Veterans General Hospital, Kaohsiung, Taiwan; ^3^Dental Laboratory Technology, Shu Zen College of Medicine and Management, Kaohsiung, Taiwan; ^4^Dental Department, Kaohsiung Municipal Siaogang Hospital, Kaohsiung, Taiwan; ^5^Laser and Additive Manufacturing Technology Center, Industrial Technology Research Institute, Taiwan; ^6^School of Dentistry and Institute of Oral Medicine, College of Medicine, National Cheng Kung University, Tainan, Taiwan; ^7^Department of biomedical engineering, Hungkuang University, Taichung, Taiwan; ^8^KSVGH Originals & Enterprises, Kaohsiung Veterans General Hospital, Kaohsiung, Taiwan

## Abstract

Segmental bony defects of the mandible constitute a complete loss of the regional part of the mandible. Although several types of customized three-dimension-printed mandible prostheses (CMPs) have been developed, this technique has yet to be widely used. We used CMP with a pressure-reducing device (PRD) to investigate its clinical applicability. First, we used the finite element analysis (FEA). We designed four models of CMP (P1 to P4), and the result showed that CMP with posterior PRD deployment (P4 group) had the maximum total deformation in the protrusion and right excursion positions, and in clenching and left excursion positions, posterior screws had the minimum von Mises stress. Second, the P4 CMP-PRD was produced using LaserCUSING from titanium alloy (Ti-6Al-4V). The fracture test result revealed that the maximum static pressure that could be withstood was 189 N, and a fatigue test was conducted for 5,000,000 cycles. Third, animal study was conducted on five male 4-month-old Lanyu pigs. Four animals completed the experiment. Two animals had CMP exposure in the oral cavity, but there was no significant inflammation, and one animal had a rear wing fracture. According to a CT scan, the lingual cortex of the mandible crawled along the CMP surface, and a bony front-to-back connection was noted in one animal. A histological examination indicated that CMP was significantly less reactive than control materials (*p* = 0.0170). Adequate PRD deployment in CMP may solve a challenge associated with CMP, thus promoting its use in clinical practice.

## 1. Introduction

The segmental bony defect of the mandible is a complete loss of the regional part of the mandible. The possible reasons are from infection, mandibular osteomyelitis, tumor resection, or comminuted mandibular fracture. After encountering a large-scale mandible defect, it is very important to reconstruct it immediately [1, 2]. Many reconstruction methods in the past have been mentioned to restore the appearance and normal function of the mandible. These methods include reconstruction plate, microvascular fibula free flap, iliac bone graft, costochondral rib bone graft, and alloplastic prosthesis [3, 4]. Reconstruction plates are widely used to restore mandibular continuity defects [5–9], and they can be used alone as a space maintainer or skeleton or provide as a framework for bone graft fixation [5, 10]. However, there is a clinically 5 to 47% chance that the mandibular reconstruction plate will need to be removed later due to infection, osteoradionecrosis, or partial loss of covering flap [11–15]. However, when the extent of the segmental mandible defect is too large or the composite soft tissue defect is too complicated, the use of fibula osteocutaneous flap is a standard treatment. The advantages are the long vascular pedicle, segmented blood supply, and composite tissue availability, and design with multiple bends can be performed to reconstruct the continuity of the mandible and provide a relatively sufficient amount of bone, which may be able to carry the dental implant [9]. However, donor-site morbidity, a lengthy operation, scar constructure, and suboptimal cosmetic results are possible disadvantages [8, 9].

The development of computer-aided design (CAD) and computer-aided manufacturing (CAM) systems allows us to use it in the preoperative surgical planning and the production of customized metal implants [2, 5] and has recently been introduced to the field of maxillofacial bone reconstruction [16]. Customized three-dimensionally printed mandible prostheses (CMP) have successfully been used for the reconstruction segmental defect of the mandible. The advantage of CMP is that it can be designed according to the defect size and morphology [17] and can be fitted accurately in the defective site without interference after computer simulation [18].

There have been several in vivo animal experiments investigating the customized mandible implant [19, 20], but most of them were limited to the titanium shelf with artificial bone substitutes. Hong et al. [21] studied rabbits with total customized mandible implant that showed higher and faster recovery rates of their daily food intake amount and higher screws intact rate than those treated with five-hole miniplates without bone grafts. In human studies, several case reports or series used customized mandible implant which behaved as mesh or framework structures with or without artificial bone substitutes [4, 22]. Some researchers [1, 23–25] further used titanium implant with premounted dental implants for mandible reconstruction. Polyether ether ketone (PEEK) is a printable and biocompatible material to human bones [26, 27] and Cheng et al. [28] proposed that the optimized PEKK bone analog model creates more normal stress-strain trajectories than the fibular graft model and likely provides better functional and cosmetic outcomes for mandible reconstruction in an in vitro study.

Although several types of CMP have been published in the literatures of animal [29, 30] and human [1, 4, 25, 31, 32] in the past, this technique has not yet been widely used and lacks complete follow-up results. The reasons might be from the difficulty in performing surgery, and it takes too much time on the cost of design and production. However, the complex chewing state in the oral cavity may also be the cause of the failure of the operation. The temporomandibular joint system allows rotation and translation of the mandible, and the major masticatory muscles, namely the superficial masseter, the deep masseter, the anterior temporalis, the middle temporalis, the posterior temporalis, the lateral pterygoid, the medial pterygoid, and the digastric muscles, attached to the mandible, provide multidirectional strength. These numerous structures were combined to create a complex biomechanical environment. In addition, the masticatory movement cannot be completely rested even during sleep [33].

In view of the above complex factors, besides structural rigidity considerations, the design of CMP should also include the design of pressure-reducing device (PRD). Stress shielding effect means the bone plate bears most of the stress after fixation, but the bone must also share part of the stress to provide stimulation for bone growth to promote posttraumatic osteogenesis [34]. A lack of this stress shielding may cause bone resorption, deficient callus formation, delayed union, late implant failure, and nonunion [35]. Moghaddam et al. [36] proposed a long-span titanium plate with stepped strain-releasing structure, and the results for stress distribution on the reconstructed mandible during the released state closely match that of a healthy mandible. Therefore, the design of PRD could possibly reduce the concentration of masticatory stress and keep the strain within an appropriate range which is required in the newly formed bone tissue by allowing controlled interfragmentary movements along the bone's axial direction [34].

Computer-aided engineering (CAE) describes the utilization of a computer and its software for the purposes of designing, analyzing, and generating products and methods, and finite element analysis (FEA) is one of the numerical techniques that simulate the mechanical aspect of a structure under load [37]. FEA has been widely used to predict the effect of stress on biomaterials and its surrounding structure for the last 30 years [7, 38–40]. Although FEA has been widely used in the past to evaluate the force applied by the jaw chewing to the various implants [41–43], it is mostly applied in the clenching condition. In this research, we evaluated four different chewing positions of the mandible (clenching, protrusion, right excursion, and left excursion) and the vector changes of the corresponding masticatory muscles. Using this model, we studied whether the deployment of different positions of PRD (negative, front, rear, and front-rear) will affect the changes in the stress and strain of CMP. Based on our findings, we will conduct the experimental test setup according to ISO 14801 to confirm that if the fatigue and mechanical properties meet the clinical expectations, we will further execute this result on living organisms. In this animal experiment, we chose the Lanyu pigs because the shape and size of its mandibles are more similar to those of actual humans compared with the smaller rats, rabbits, and minipigs.

## 2. Materials and Methods

### 2.1. Generation of the Geometric Model

**Figure 1:**
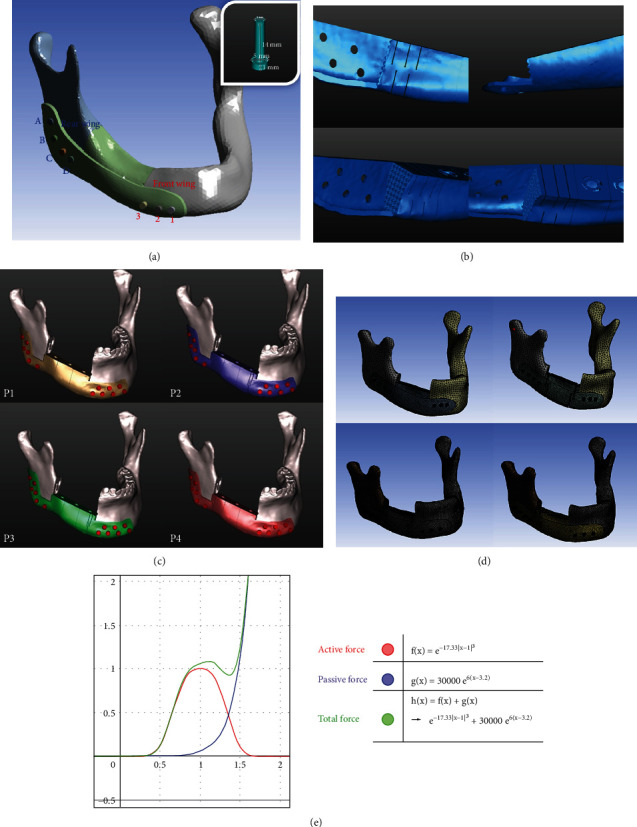
(a) The CMP, which includes the main body, a front wing, and a rear wing, was fixed to the remaining mandible. The front wing was affixed with three screws (Coded as 1, 2, and 3), and the rear wing was affixed with four screws (Coded as A, B, C, and D). (b) The PRD at either end of the main body contains three to five parallel hollow structures with terminal hollow cylinders at each line that serve as stress breakers. (c) Four CMP-PRDs: P1 without a PRD, P2 with anterior and posterior PRDs, P3 with an anterior PRD, and P4 with a posterior PRD. (d) The models were meshed with tetrahedral structural solid elements. (e) The total force equation by Hill et al. was used to calculate the total force of the muscle obtained as the sum of the active and passive forces.

Two digital mandible solid models were constructed using data from computed tomography (CT) scans of a normal male patient and one subject (Animal 4) of our animal experiments. The preoperative cranial-maxillofacial CT scan (slice thickness, 76 *μ*m) (KODAK 9000 3D® CBCT (Carestream Health, Inc.) was performed with a stable occlusion. The Digital Imaging and Communications in Medicine (Dicom) files were stored and then imported into the Mimics software (version 18.0; Materialize, Leuven, Belgium) for the three-dimensional reconstruction (Version 10.01, Materialise, Inc.). The defect region was defined in the unilateral mandibular body from the first premolar to the second molar. As shown in Figure 1(a), the CMP was designed to include the main body, the front, and the rear wing for fixation to the remaining mandible. The part of the mandible defect was designed according to the contralateral shape with mirroring function using the Mimics software. The PRD at the either end of the main body contains three to five parallel hollowed-out structures with terminal hollow cylinders at each line which play as a stress-breaker structure (Figure 1(b)). The thickness of the parallel hollow structures is 0.3 mm, and the diameter of the cylindrical hollowed-out structure is 0.4 mm. This PRD structure was designed to reduce the concentration of stress in the connection between the CMP and the mandible.

### 2.2. Establishment of Finite Element Model

FEA is the simulation of a physical phenomenon using a numerical mathematical technique [44]. In this study, ANSYS Workbench (Swanson Analysis Systems Co., Houston, TX, USA) was used for simulation. We made some modifications to the design of the model to facilitate subsequent analysis and processing. In order to achieve better stability in clinical practice, it is usually hoped that the fixed screws can be configured in a cross-orientation to provide fixation in different directions as shown in Figure 1(c). However, the purpose of this experiment mainly was to study the functional role of the PRD structure at different positions, and we simply designed the configuration of the fixing screws as shown in Figure 1(a). The front wing was fixed with 3 screws (Codes 1, 2, and 3), and the rear wing part was fixed with 4 screws (Codes A, B, C, and D). The diameter of the screw is 3 mm, the length of the screw is 14 mm, the diameter of the screw head is 5 mm, and the height is 2 mm. We further simplified the structure of the teeth to reduce the running time of our computer. As shown in Figure 1(b), the contact surface between the CMP-PRD and the cutting margin of the mandible has densely protruded structures to increase its friction. But we also ignored this structure while performing FEA. On the platform of the CMP-PRD, we also designed structures where commercially available dental abutments can be locked in, but it is not in the scope of this research. Finally, we designed four different forms of CMP, which included P1 without PRD, P2 with anterior and posterior PRD, P3 with anterior PRD, and P4 only with posterior PRD (Figure 1(c)).

**Figure 2:**
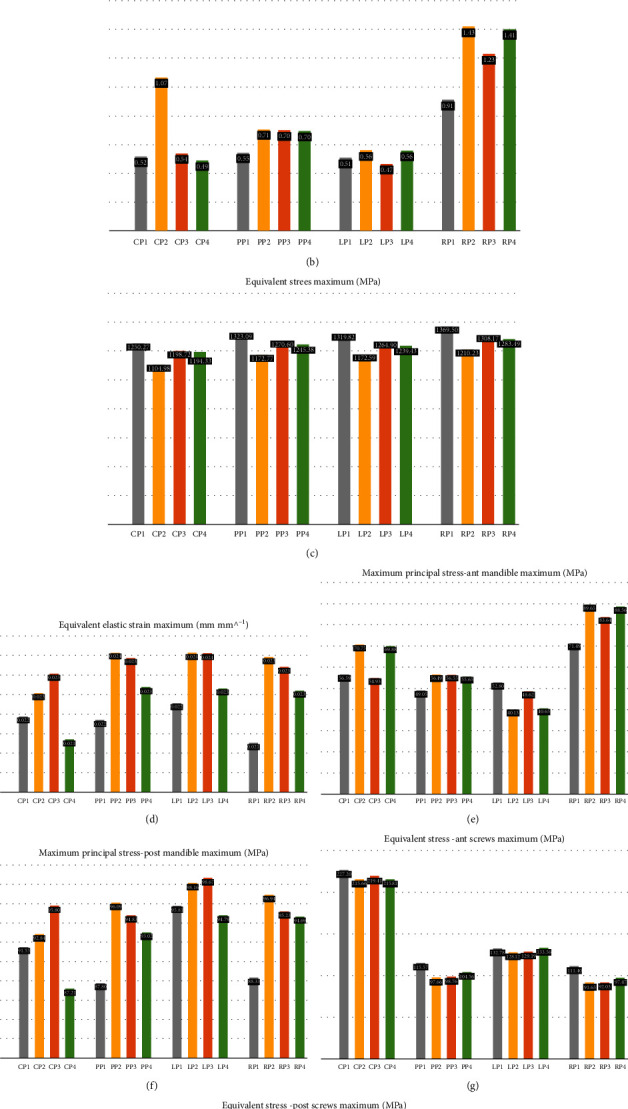
Level of total deformation, strain energy, von Mises stress, von Mises equivalent strain, and maximum principal stress of whole components, CMP-PRD (P1, P2, P3, and P4), retentive screws, anterior, and posterior mandible by FEA under the clenching (CP1-CP4), protrusion (PP1 to PP4), and left (LP1 to LP4) and right excursion (RP1 to RP4) conditions. (Supplementary material Figure 3A to 3H showed higher resolution).

**Figure 3:**
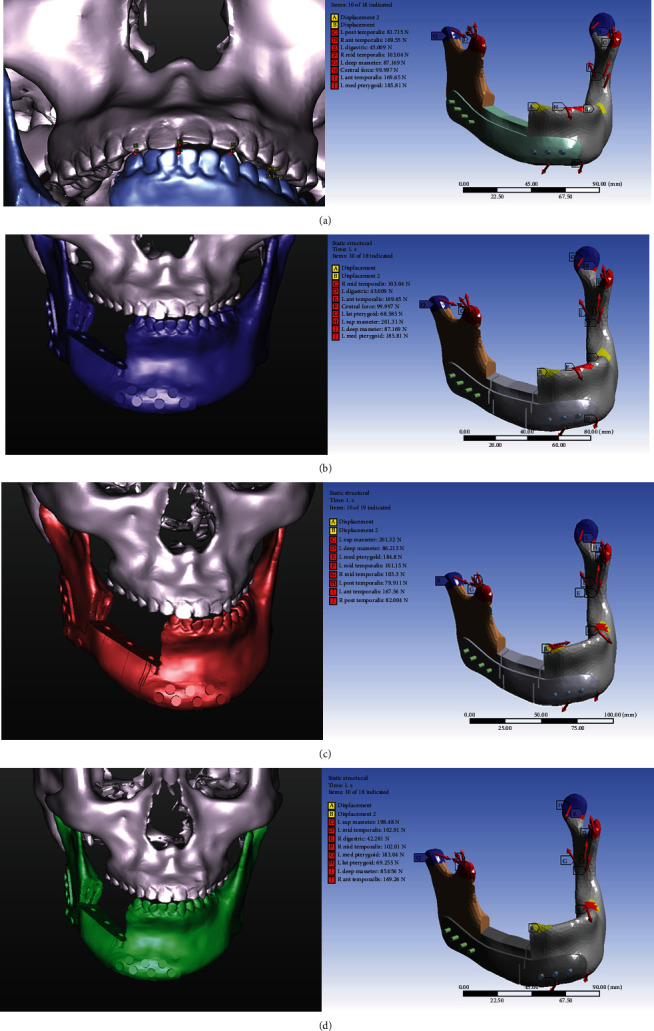
Calculated muscle vectors based on the results of 3D simulation in four different situations in mastication: (a) clenching, (b) protrusion, and (c) left and (d) right excursions. These calculated muscle vectors were applied in FEA to set the boundary condition.

Afterward, the postoperative mandible, four different forms of CMP-PRD (P1, P2, P3, and P4), the retention screws on the front wing (Codes 1, 2, and 3), and the ones on the rear wing (Codes A, B, C, and D) were imported to ANSYS Workbench for simulation. The same design method was also applied to the animal model (Figure 2). These models were meshed using quadratic formulation, second-order, full-integration, tetrahedral structural solid elements, and the average values of the aspect ratio and skewness in the human model were 5.34 and 0.55, respectively. In the animal model, the average values of aspect ratio and skewness were 5.27 and 0.51, respectively. The verification procedure in the present study focused on mesh-independent grid development [45, 46]. Convergence studies were conducted to whole components, CMP-PRD, anterior and posterior retentive screws for the optimum size of elements, and mesh density, and the variability of results was controlled at <5% for models with different element sizes (supplementary material Table S1, S2 and Figures 1 and 3). The selected numbers of mesh element and node are shown in Table 1.

Bond-type connections were applied between the interface of retentive screws and mandible. Friction-type connections (friction coefficient = 0.3) [47–49] were applied to the interface between the mandible and its corresponding CMP-PRD. Bujtar et al. [50] stated that a nonlocking plate-screw interface allowed each screw to transmit a preload force, creating a resting tension between the plate structure and bone, and it can be set as a frictional mode for practical simulation. In our study, friction-type connections (friction coefficient = 0.5) were applied to the interface between the retentive screws and the front and rear wings [51–53]. Mandible bones, CMP-PRD, and retentive screws were defined with linear elastic and isotropic properties. All elastic modulus and Poisson's ratio values were adopted from the relevant literature [54–56] as shown in Table 2.

### 2.3. Boundary Conditions and Computation of Muscle Forces

In Figure 3, the top surfaces of two condyles were fully restrained to prevent the rigid-body displacement of the mandible (purple patch). Displacement in the vertical direction of corresponding occlusal contacts was constrained over the left mandibular molar and right mandibular first premolar region (yellow patch).

Because the mastication activity of the jaw is a multidirectional movement, in this study, we analyzed the masticatory muscles vectors acting on the mandible in four different conditions, including clenching, protrusion, and right and left excursions in the human model. Because the segmental mandibular resection was performed, a reduced biting force of 300 N in the vertical direction in the contralateral molar region and 150 N in the bilateral canine and incisal region was chosen [57, 58]. The extensive resection of the mandible meant that right masseter muscles and medial pterygoid muscles were stripped of the angle of the mandible and transected; we ignored these two muscles in our study. When the mandible moves in different directions, the attached muscles and length will also change. To determine the remaining muscle forces and vectors of the defected mandible, the force-length relationship theory of skeletal muscle is our main reference basis. This theory was derived based on Hill's ground-breaking studies in isolated frog muscles [59] and was used to develop theories of the mechanisms of skeletal muscle contraction by Dr. Huxley [60]. Anderson in Stanford University further digitized the formula and published it in their literature [61]. Hill's formula proposed that as the length of the muscle changes, the total force of the muscle which includes active force and passive force changes will change accordingly (Figure 1(e)).

In our study, seven pairs of major masticatory muscles, namely the superficial masseter, the deep masseter, the anterior temporalis, the middle temporalis, the posterior temporalis, the lateral pterygoid, the medial pterygoid, and the digastric muscles with the absence of right masseter and right medial pterygoid muscles, were included and investigated in this study. The original magnitudes of normal masticatory muscle forces were used and converted to force vectors based on Nelson' s work [62]. Therefore, combining Nelson's data with Hill's formula, we inferred the final muscle vectors based on the results of the 3D simulation in four different situations of mastication, including clenching, protrusion, and right and left excursions (Figure 3). These calculated muscle vectors were applied in FEA for boundary condition setting as shown in Table 3. In addition, the values of muscle forces in *X*, *Y*, and *Z* directions in clenching position in animal models are referred from the report of Langenbach et al.'s [63] as shown in Table 4.

### 2.4. Biomechanical Testing

**Figure 4:**
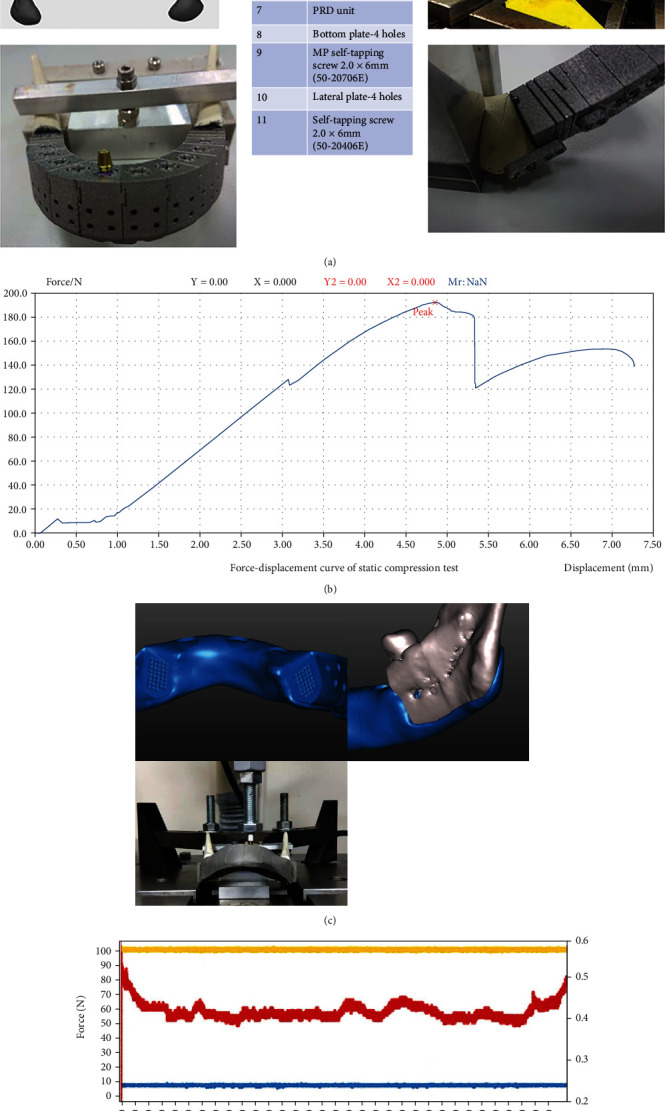
(a) Detailed configuration of the test of CMP-PRD. We selected an angle-to-angle defect area of the mandible to simulate the worst-case scenario in a clinical setting. We found an incomplete crack on the lingual side of the mandible (black triangle). (b) The fracture test result indicated that the maximum static pressure that could be withstood was 189 N. (c) A covered support structure on the lingual side of the rear wing was deployed to reduce the risk of fracture and the dummy mandible did not fall off, consistent with the aforementioned worst-case scenario. (d) Fatigue test was conducted for 5,000,000 cycles on the right mandibular canine, with the loading frequency of 3 Hz and load range of 10–100 N.

The CMP-PRD was produced from titanium alloy (Ti-6Al-4V) by the manufacturer (Industrial Technology Research Institute, Tainan branch, Taiwan), and the LaserCUSING® technique was chosen. We divided the implant into multiple standardized small units to be able to adapt to various clinical conditions. The detailed configuration is shown in the middle table of Figure 4(a). This study used the Stryker-Leibinger fixation system (Stryker-Leibinger Micro Corp., Freiburg, Germany) to simulate the real clinical situation. We used the smallest-sized bone screws (Mandible self-tapping screw; 50-20406) to fix the PRD units (symbol 7) with corresponding lateral bone plates (symbol 10) and the bottom plates (symbol 8) to the dummy mandible. The CMP units were assembled with built-in locking structures (mortise and tenon joint), bottom fixed bone plates (symbol 6), and self-tapping screws (50-20706). In our study, we selected the angle-to-angle defect area of the mandible to simulate the most severe case scenario.

The technical design of the experimental test was set up according to ISO 14801 to confirm if the fatigue and mechanical properties meet clinical expectations. The loading was conducted with servohydraulic/electric testing machines (Hung Ta, Instrument Co., LTD, Taipei, Taiwan) with an axial load platform (Figure 4(c)). Fracture tests were performed with a downward load velocity of 3 Hz on the right canine region, and the fracture force was recorded upon sample failure.

### 2.5. Mesh Sensitivity Analysis

**Figure 5:**
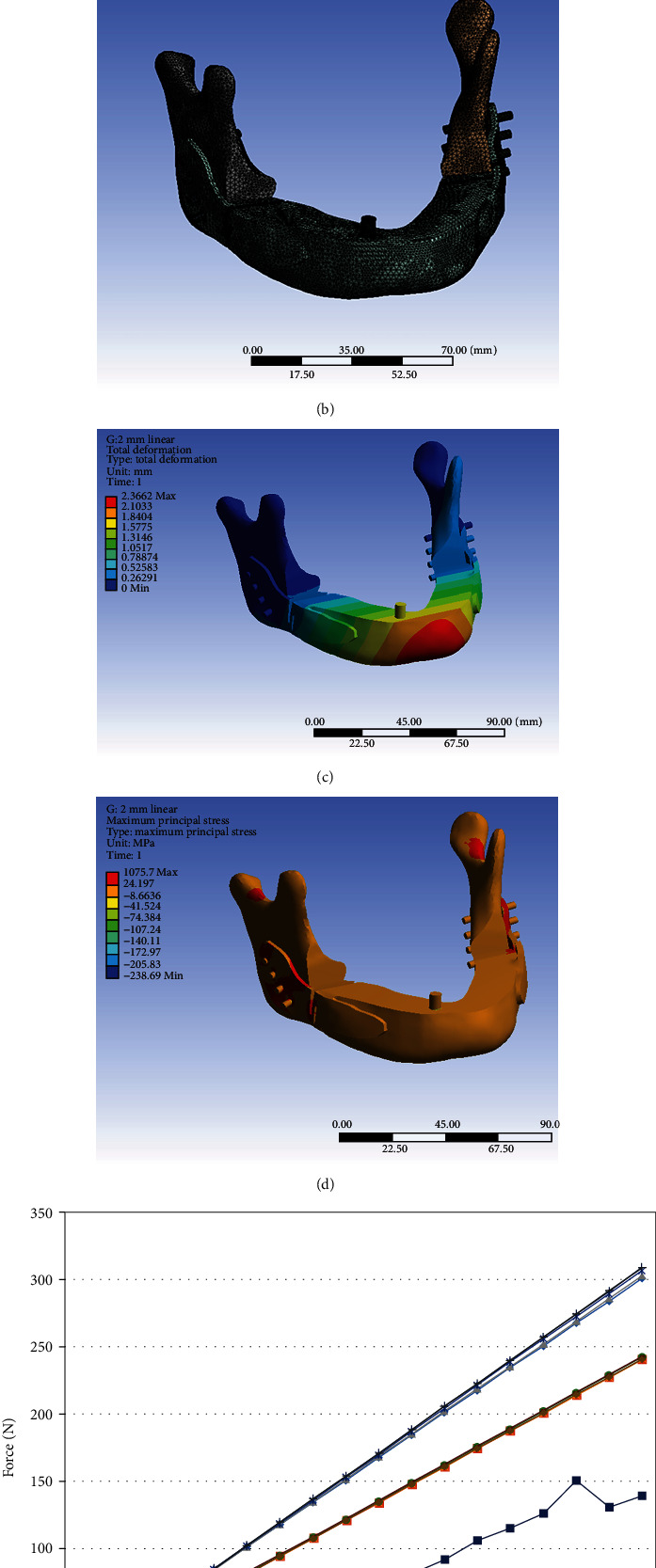
(a–d) The biomechanical test corresponding FE models were meshed with element sizes of 1.5, 2, 2.5, 2.75, and 3 mm and linear and quadratic formulation (8 and 20 nodes) under sequential vertical force (20-300 N) over the anterior CMP abutment. (e) The force-displacement values obtained from the FE models were compared to known experimental values obtained from the biomechanical test (Supplementary material Figure 5A and 5B showed higher resolution).

We rebuilt biomechanical testing corresponding to the FE model to perform the sensitivity analysis (Figures 5(a)–5(d)) and calculated MAE, MSE, and RMSE to determine the adequate size of the elements and its numerical formulation used to ensure correct results at the lowest computational cost according to previous studies [64, 65]. For all FE models, element sizes of 1.5, 2, 2.5, 2.75, and 3 mm and linear and quadratic formulation (8 and 20 nodes), respectively, were used. The force-displacement values obtained from the FE models were compared to known experimental values (Figure 4(b)).  Figure 5(e) (supplementary material Figure S5A and S5B) showed that as the size of the element decreases and its number of nodes increases (20 nodes/quadratic formulation), the difference between the force-displacement curves that were obtained from the FE models and those that were obtained experimentally is smaller. The force obtained from the experimental group for 3 mm displacement was 126.46 N. The force obtained from the FE model with same displacement was 250.73, 252.55, 255.57, 257.98, and 257.66 N for linear formulation and was 201.07, 200.99, 202.23, 202.27, and 200.94 N for the quadratic formulation with element sizes of 1.5, 2, 2.5, 2.75, and 3 mm, respectively.

We further calculated MAE, MSE, and RMSE under different mesh sizes (1.5, 2, 2.5, 2.75, and 3 mm) and formulation (linear/quadratic) according to Equations (1)–(3). In these three equations, *Y*_EXP_ are the forces that were obtained experimentally for a value of displacement *i*, *Y*_FEM_ are those forces that were obtained from the FE simulations for the corresponding values of displacement *i*, and *n* is the number of force-displacement values that were used to make the adjustment.

(1) $$\begin{array}{c} \text{MAE}=\frac{1}{n}\;\overset{n}{\underset{i=1}{\sum }}\left|Y_{i\text{EXP}}-Y_{i\text{FEM}}\right|, \end{array}$$

(2) $$\begin{array}{c} \text{MSE}=\frac{1}{n}\;\overset{n}{\underset{i=1}{\sum }}{\left(Y_{i\text{EXP}}-Y_{i\text{FEM}}\right)}^{2}, \end{array}$$

(3) $$\begin{array}{c} \text{RMSE}=\sqrt{\frac{1}{n}\;\overset{n}{\underset{i=1}{\sum }}{\left(Y_{i\text{EXP}}-Y_{i\text{FEM}}\right)}^{2}}. \end{array}$$

Table 5 showed that while the size of the elements decreased and the proposed FE models had a quadratic formulation (20 nodes), the computational cost increased and the values of MAE, MSE, and RMSE decreased. The smallest results obtained were for mesh sizes of 1.5 mm and quadratic formulation. Finally, a mesh size of 2.75 mm and a quadratic formulation were selected in our study because the values of MAE, MSE, and RMSE were reduced (59.67, 4090.02, and 63.95, respectively), and their computational costs were relatively acceptable (18.77 min) among all groups.

#### 2.5.1. Animal Model

The experiments were performed in accordance with ARRIVE guidelines 2.0, and the ARRIVE checklist was attached in the supplementary file. We followed the Council of Agriculture Executive Yuan guideline for the care and use of the laboratory guidebook in Taiwan and the suggestions in the guidebook for the care and use of laboratory animals. The protocol of the study was approved by the Commission for Animal Studies at the Institutional Review Board of Kaohsiung Veterans General Hospital, Kaohsiung, Taiwan (file number 2020-A043).

The experiments were performed by using 5 male Lanyu pigs with an average age of 4 months and an average weight of 20 kg. The animals were obtained from a certified breeding company (Livestock Research Institute, Council of Agriculture, Tainan, Taiwan). The animals were kept in the Experimental Center of the Medical Faculty and were allowed to adapt to the environment one week prior to surgeries. At the beginning of the study, all animals underwent a physical examination by a veterinarian and were found to be healthy, and the identification of the animals was enabled by ear tag. The animals were placed in appropriate single space with straw bedding, and fresh water was available ad libitum. Prior to surgical interventions and the postoperative healing period until sacrifice, the animals were fed mashed bran.

#### 2.5.2. Anesthesia

All interventions were performed in general anesthesia under the surveillance of a veterinarian. General anesthesia was induced by intravenous injection of 1 mg per kg body weight midazolam which has successfully been used as a preanesthetic tranquilizer. Endotracheal intubation was followed carefully because swine are one of the more difficult species to intubate, and improper technique can result in significant trauma, e.g., laryngeal rupture or passage of the endotracheal tube in the subcutaneous space. Maintenance of the general anesthesia was achieved by 3-5% isoflurane under mechanical ventilation. For infection prophylaxis, 15 mg per kg body weight of enrofloxacin 5 mg/kg were injected intravenously. Jaw tones were assessed throughout the procedure, and the analgesia was performed by administration of ketoprofen 5 mg per kg body weight intravenously.

#### 2.5.3. Surgical and Postsurgical Procedures

**Figure 6:**
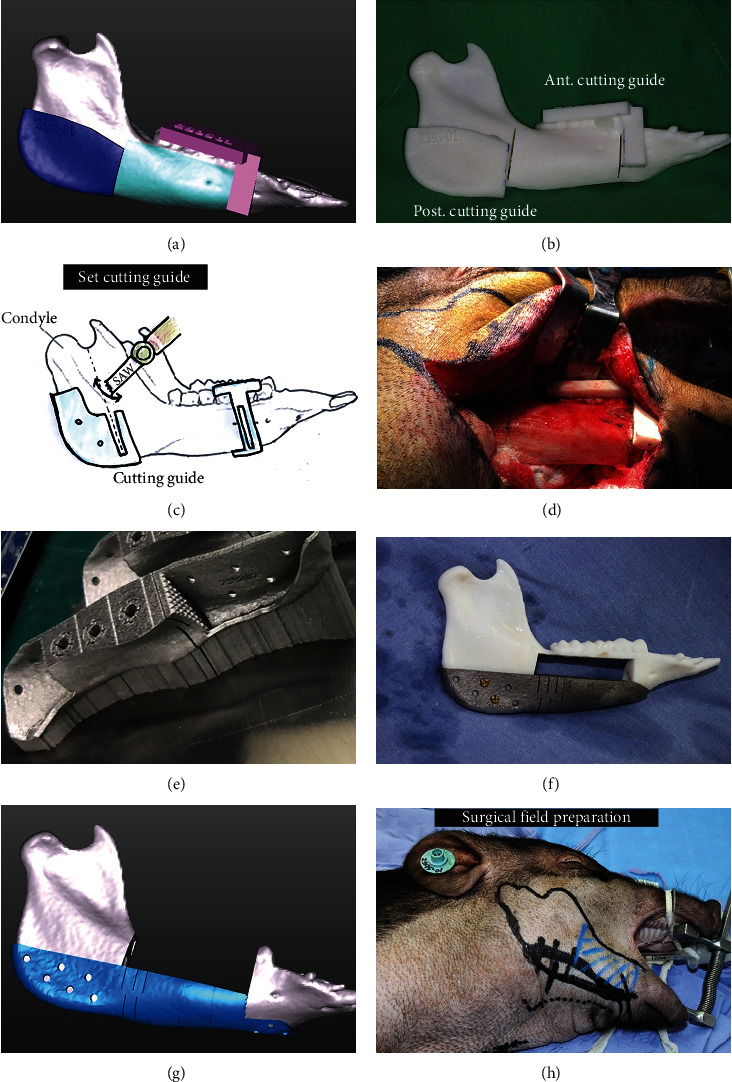
(a) Individualized templates for osteotomy. (b) Light-cured 3D-printed surgical templates. (c) The mandibular body between the right first premolar and the third molar was resected using 5 mm-wide reciprocating saw blades. (d, h) The mandibular corpus was prepared and fully exposed through submandibular skin incision. The templates were fixed on the mandible angle posteriorly and the tooth surface anteriorly. (e–g) The CMP-PRD was fabricated from pure titanium using the LaserCUSING technique.

This experiment method was mainly carried out with reference to the experimental procedures of Markwardt et al. [30]. One week prior to surgery, the computed tomography (CT) scan of the mandible for each of the animals was performed, and a three-dimensional model of the mandible was created. The cutting planes were defined in the region distal of the first premolar and mesial of the third molar in the right mandible. Thus, a fragment of the right mandible containing the second and third premolar as well as the first and second molar was planned to be removed. Based on this model, the templates for the osteotomy and the mandibular implant were created individually for each animal (Figure 6(a)). Subsequently, the light-curing 3D-printed surgical templates were produced by the Research Development Innovation Center in Kaohsiung Veterans General Hospital (Figure 6(b)). The CMP-PRD was produced from titanium alloy by the manufacturer (Industrial Technology Research Institute, Tainan branch, Taiwan), and in order to produce a shape-identical titanium implant resembling the removed part of the mandible, the LaserCUSING® technique was chosen (Figures 6(e) and 6(f)).

**Figure 7:**
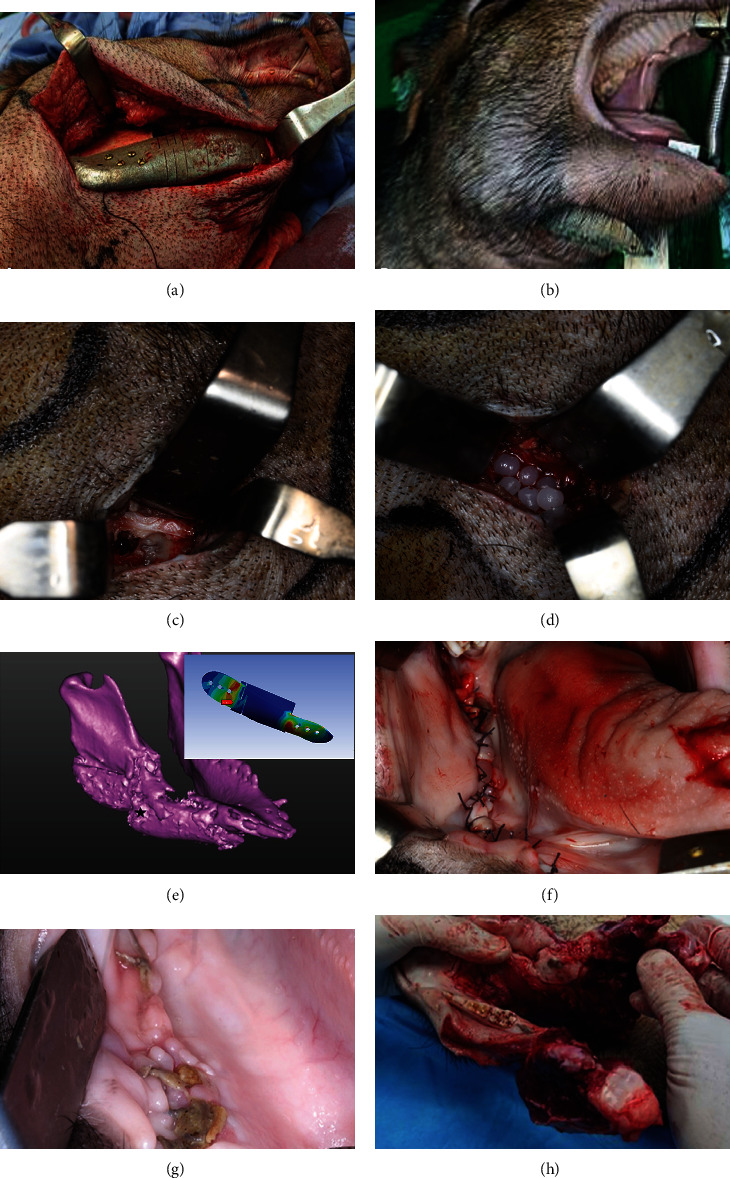
(a) The CMP-PRD was inserted from the submandibular exposure wound. (b) The CMP was loose, and the front end of the CMP penetrated Animal 1's skin. (c, d) On the left side of the mandible, HDPE was placed as a control material for histopathological comparison. (e) Animal 4: a complete fracture line was observed over the connection between the CMP and rear wing, and the anterior segment was displaced downward. (f) The vestibular wound was primarily closed with 3-0 Vicryl after appreciable tissue release and removal of sharp bones at the front and rear mandible. (g) Fair healing of intraoral wounds 1 month later. (h) The CMP remained in the original position after scarring.

Following the induction of general anesthesia, a transoral incision of the papillary margin from the canine tooth to the ascending ramus of the right mandible was performed. Subsequently, full-thickness mucoperiosteal flaps on the vestibular and lingual site were elevated (Figure 6(h)). Through submandibular skin incision, the corpus of the mandible was prepared and fully exposed. The individually prepared surgical templates were fixed on the mandible angle posteriorly and the tooth surface anteriorly (Figure 6(d)). According to the designed cutting plane, the part of the mandible between the first premolar and the third molar was resected using 5 mm width reciprocating saw blades (Figure 6(c)). After copious irrigation with normal saline, the CMP was inserted from the submandibular exposure wound (Figure 7(a)). Because the CMP was designed to have a rough structure at the interface with the bone (Figure 1(b)), it must have a squeezing force when fixing it. Finally, the front and rear wings were fitted and fixed with three and six screws (8 mm length), respectively, to the corresponding mandible. The vestibular wound was primarily closed with 3-0 Vicryl after appreciable tissue release and sharp bone removal at the front and rear mandibles. The submandibular incision was sutured in layers, and a surgical drain was inserted to prevent free fluid and blood accumulation (Figure 7(f)).

On the opposite side of the mandible, we incised the skin down to the surface of the ramus and created a 1 × 1-centimeter-size cavity. We placed HDPE as controlled materials (high-density polyethylene (HDPE), Bormed HE7541-PH, Lot. 2400005143, FDA Drug Master File number: DMF18351) for histopathological comparison and performed a two-layered closure (Figures 7(c) and 7(d)).

Postoperative care includes daily intramuscular injection of enrofloxacin 5 mg/kg and ketoprofen 5 mg/kg once a day for one week. We used betadine to clean and disinfect surgical wounds and oral chlorhexidine bactericidal syrup to clean the oral cavity twice a day for one consecutive week.

Two weeks after the operation, intravenous general anesthesia was induced by intramuscular injection of 1 mg/kg midazolam and 10 mg/kg ketamine, and the CMPs were examined regarding their clinical stability and signs of inflammation, e.g., suppuration, swelling, or exposure of the implant. Maintenance of the anesthesia was achieved by administering half of the initial dose intramuscularly. During this time, we also cleaned the oral wounds and removed the oral and skin stitches. Two months later, we took computer tomography of each animal to assess the location of the CMPs. According to the study protocol, the animals were sacrificed after 3 months.

#### 2.5.4. Euthanasia and Specimen Retrieval

At the end of each observation period, the animals were euthanized, and heart exsanguination was practiced under deep anesthesia, intramuscular injection of Zoletil 50 5 mg/kg, xylaine 2 mg/kg, atropine 0.03 mg/kg plus ketoprofen 2 mg/kg, and following 3% isoflurane. Following gross necropsy, the implanting sites were collected, fixed, and preserved in 10% neutral buffered formalin for subsequent histopathology examination. The specimens were trimmed, embedded, sectioned, and H&E stained, followed by microscopy examination.

This histopathological procedure was to evaluate the local effects after implanting, and this study was in accordance with the ISO 10993-6: 2016, Biological Evaluation of Medical Devices—Part 6: Tests for Local Effects after Implantation (Master Laboratory Co., Ltd. Hsinchu, Taiwan).

### 2.6. Statistical Analysis

We compared the inflammatory scores between the CMP and controls which include polymorphonuclear leukocytes, lymphocytes, plasma cells, macrophages, giant cells, and necrosis and the average scores of neovascularization, fibrosis, and fatty infiltration in accordance with the ISO 10993-6: 2016. The analysis of data was carried out using GraphPad Prism version 9 for Windows, and a paired *t* test with estimation plots was used. The left axis is scaled to show the data, and the right is scaled to show the effect size and its confidence level. A probability value of less than 0.05 was regarded as statistically significant.

## 3. Results

### 3.1. Changes in Muscle Vector and Strength in Different Positions in Human and Animal Models

**Figure 8:**
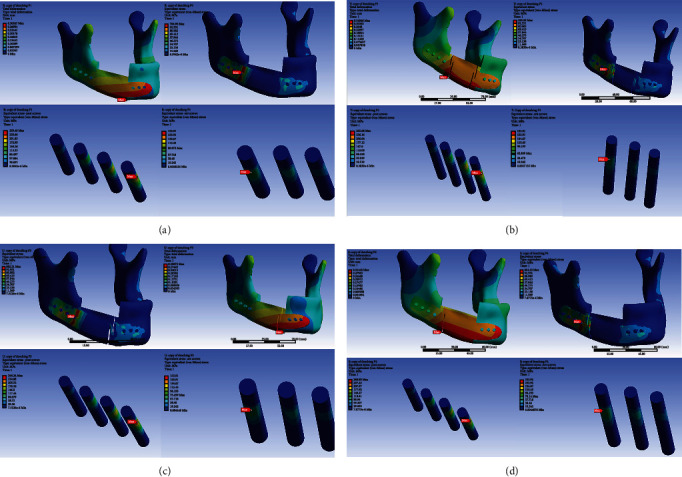
Total deformation and von Mises stress of whole components, CMP-PRD (P1, P2, P3, and P4) and individual retentive screws by FEA under (a–c) the clenching condition and (d–f) the four conditions (clenching, protrusion, and right and left excursion conditions).

Table 3 show the values of total forces, strength, muscle weight, and muscle forces in *X*, *Y*, and *Z* directions calculated as referred from Nelson [62] in clenching, protrusion, right excursion, and left excursion positions in the human model. A loading (300 N) on the left mandibular first molar and two loadings (150 N) on the bilateral lower canine were designated in clenching position as shown in Figure 3(a). One loading condition (100 N) was designated on the lower central incisor in protrusion position as shown in Figure 3(b). Table 3 show the values of muscle forces calculated for postoperative patients in the right excursion position. One loading condition (300 N) was designated on the left molar as shown in Figure 3(c).  Figure 3(d) shows the values of muscle forces calculated for postoperative patients in the left excursion position, and two loading conditions were designated: loading (300 N) on the left molar and loading (150 N) on the right lower canine.  Figure 8 shows total deformation and von Mises stress of whole components, CMP-PRD (P1, P2, P3, and P4) and individual retentive screws by FEA under the four conditions.

**Figure 9:**
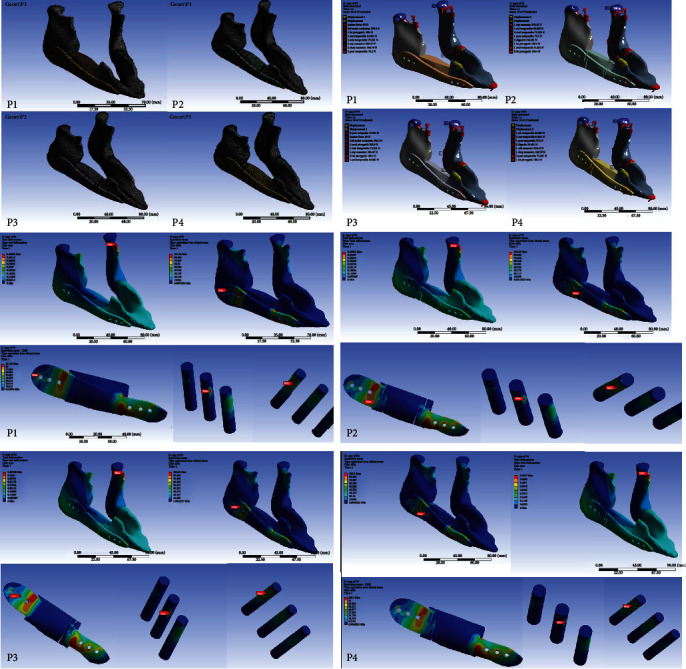
The animal models were meshed with tetrahedral structural solid elements, and the calculated muscle vectors under clenching were applied in FEA to set the boundary condition. Total deformation, von Mises stress, and principal stress of whole components, CMP-PRD (P1, P2, P3, and P4), anterior and posterior retentive screws, and mandible were analyzed by FEA under the clenching condition.

Table 4 shows the values of muscle forces in *X*, *Y*, and *Z* directions in clenching position in an animal model as referred from Langenbach et al. [63] A loading (250 N) on the left mandibular first molar and the other loading (90 N) on the mandibular central incisor were designated in clenching position [66] as shown in Figure 9.

### 3.2. Total deformation, von Mises Equivalent Strain, von Mises Stress, and Maximum Principal Stress of Whole Components in Four Positions in the Human Model

Figure 2 (supplementary material Figure S3A to S3H showed higher resolution) and Table 6 show the level of total deformation, strain energy, von Mises equivalent strain, von Mises stress, and maximum principal stress of whole components, CMP-PRD (P1, P2, P3, and P4) and individual retentive screws by FEA under the clenching (CP1-CP4), protrusion (PP1 to PP4) and left (LP1 to LP4) and right (RP1 to RP4) excursion conditions.

#### 3.2.1. Total Deformation

In clenching position, the CMP-PRD with the maximum total deformation is P2, followed by P4, P1, and P3; in protrusion and right excursion positions, P4 had the maximum total deformation, and P1 had the minimum total deformation; in the left excursion position, P1 had the maximum total deformation, and P4 had the minimum total deformation (Figure 2(a), supplementary material Figure S3A).

#### 3.2.2. Strain Energy

The CMP-PRD with the maximum strain energy is P2 in all four conditions. In clenching position, the CMP-PRD with the maximum strain energy is P2 (1.702 mJ), followed by P3, P1, and P4 (0.491 mJ); in protrusion and right excursion positions, P1 had the minimum strain energy (Figure 2(b), supplementary material Figure S3B).

#### 3.2.3. von Mises Equivalent Strain

In clenching position, the CMP-PRD with the maximum von Mises equivalent strain is P3 (0.023 mm/mm), followed by P2, P1, and P4 (0.021 mm/mm); in other three conditions, the P2 with the maximum von Mises equivalent strain, followed by P3, P4, and P1 (Figure 2(d), supplementary material Figure S3D).

#### 3.2.4. von Mises Stress of Whole Components, Anterior and Posterior Screws

In Figure 2(c) and supplementary material Figure S3C, in all four conditions, the CMP-PRD with the maximum von Mises stress is P1, and the one with the minimum von Mises stress is P2. In protrusion, left, and right excursion positions, P4 had the second lowest von Mises stress results (1245, 1239, and 1283 MPa). In all positions, anterior screws had the maximum von Mises stress (227.20, 115.57, 132.78, and 111.40 MPa) in the P1 group, followed by anterior screws in P4, P3, and P2 groups, in protrusion, left, and right excursion positions (Figure 2(g), supplementary material Figure S3G). In Figure 2(h) (supplementary material Figure S3H), in clenching and left excursion positions, posterior screws had the maximum von Mises stress (459.75 and 483.62 MPa) in the P3 group, followed by posterior screws in P2, P1, and P4 groups. In protrusion and right excursion positions, posterior screws had the maximum von Mises stress (470.43 and 477.06 MPa) in the P2 group, followed by posterior screws in P3, P4, and P1 groups. In addition, in four positions and four CMP-PRDs, the anterior screws with the maximum von Mises stress are Code 3, and the posterior screws with the maximum von Mises stress are Code D.

#### 3.2.5. Maximum Principal Stress of Anterior and Posterior Mandible Components

In clenching and right excursion positions, the anterior mandible had the maximum principal stress (70.75 and 89.65 MPa) in the P2 group, so as the protrusion position (56.55 MPa) in the P3 group and left excursion position (52.80 MPa) in the P1 group, respectively. In protrusion and right excursion positions, the anterior mandible had the minimum principal stress (49.05 and 71.49 MPa) in the P1 group, so as the clenching position (54.93 MPa) in the P3 group and left excursion position (40.13 MPa) in the P2 group, respectively (Figure 2(e), supplementary material Figure S3E). In Figure 2(f) (supplementary material Figure S3F), in protrusion, left, and right excursion positions, the posterior mandible had the maximum principal stress (96.09, 98.16, and 96.94 MPa) in the P2 group; in clenching position, the posterior mandible had the maximum principal stress (95.80 MPa) in the P3 group. In protrusion and right excursion positions, posterior mandible had the minimum principal stress (87.81 and 88.33 MPa) in the P1 group, so as the clenching position (87.21 MPa) in the P4 group and left excursion position (98.67 MPa) in the P3 group, respectively.

### 3.3. Total Deformation, von Mises Equivalent Strain, von Mises Stress and Maximum Principal Stress of Whole Components in Clenching Position in the Animal Model

**Figure 10:**
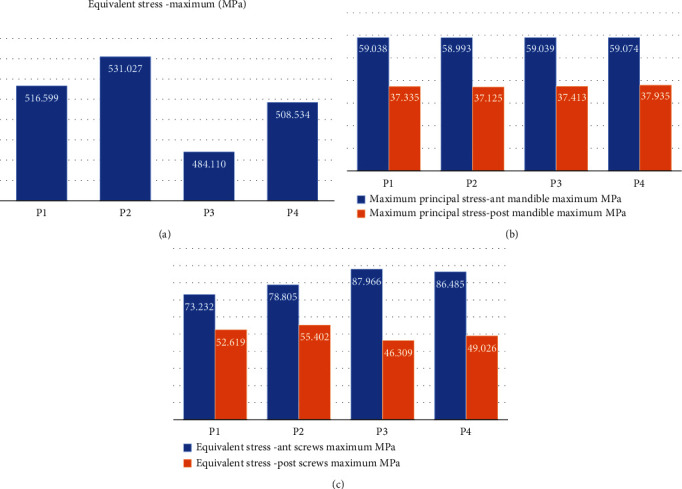
The level of von Mises stress of (a) whole components, (b) anterior and posterior retentive screws, (c) maximum principal stress of anterior and posterior mandible with different CMP-PRDs (P1, P2, P3, and P4) was analyzed by FEA under the clenching condition in the animal model. (Supplementary material Figure 4A, 4B and 4C showed higher resolution).

Figure 10 (supplementary material Figure S4A to S4C showed higher resolution) and Table 7 show the level of total deformation, strain energy, von Mises equivalent strain, von Mises stress, and maximum principal stress of whole components, CMP-PRD (P1, P2, P3, and P4), and individual retentive screws by FEA under the clenching condition.

#### 3.3.1. von Mises Stress of CMP-PRD and Its Retentive Screws

In Figure 10(a) (supplementary material Figure S4A), the CMP-PRD with the maximum von Mises stress is P2, followed by P1, P4, and P3 (531.03, 516.60, 508.53, and 484.11 MPa). In Figure 10(c) (supplementary material Figure S4C), anterior screws had the maximum von Mises stress in the P3 group, followed by anterior screws in P4, P2, and P1 groups (87.97, 86.49, 78.81, and 73.23 MPa). Posterior screws had the maximum von Mises stress in P2 group, followed by anterior screws in P1, P4, and P3 groups (55.40, 52.62, 49.03, and 46.31 MPa).

#### 3.3.2. Maximum Principal Stress of Anterior and Posterior Mandible Components

The anterior mandible had the maximum principal stress in the P4 group, followed by anterior screws in P3, P1, and P2 groups (59.07, 59.04, 59.04, and 58.99 MPa). The posterior mandible had the maximum principal stress in the P4 group, followed by anterior screws in P3, P1, and P2 groups (37.94, 37.41, 37.34, and 37.13 MPa) (Figure 10(b), supplementary material Figure S4B).

### 3.4. Fracture and Fatigue Test Results

The fatigue test was conducted for five million cycles on the right mandibular canine as the static loading test to clarify the fatigue cracking behavior and fatigue strength. The loading frequency was 3 Hz, and the load range was set to be 10 N-100 N (Figure 4(d)). The dummy mandible has no falling off or displacement, which is in line with the worst clinical situation (Figure 4(a)). The fracture test result showed that the maximum static pressure withstand value was 189 N (Figure 4(b)), and we found an incomplete crack line on the lingual side of the mandible (black triangle, Figure 4(a)). To solve this phenomenon that may be encountered clinically, we extended a covered support structure on the lingual side of the rear wing to reduce the risk of fracture caused by lateral stress (black triangle indicate, Figure 4(c)), and it also passed the fatigue test in another fatigue test (Figure 4(e)).

### 3.5. Animal Experiment

#### 3.5.1. Clinical Follow-Up

**Figure 11:**
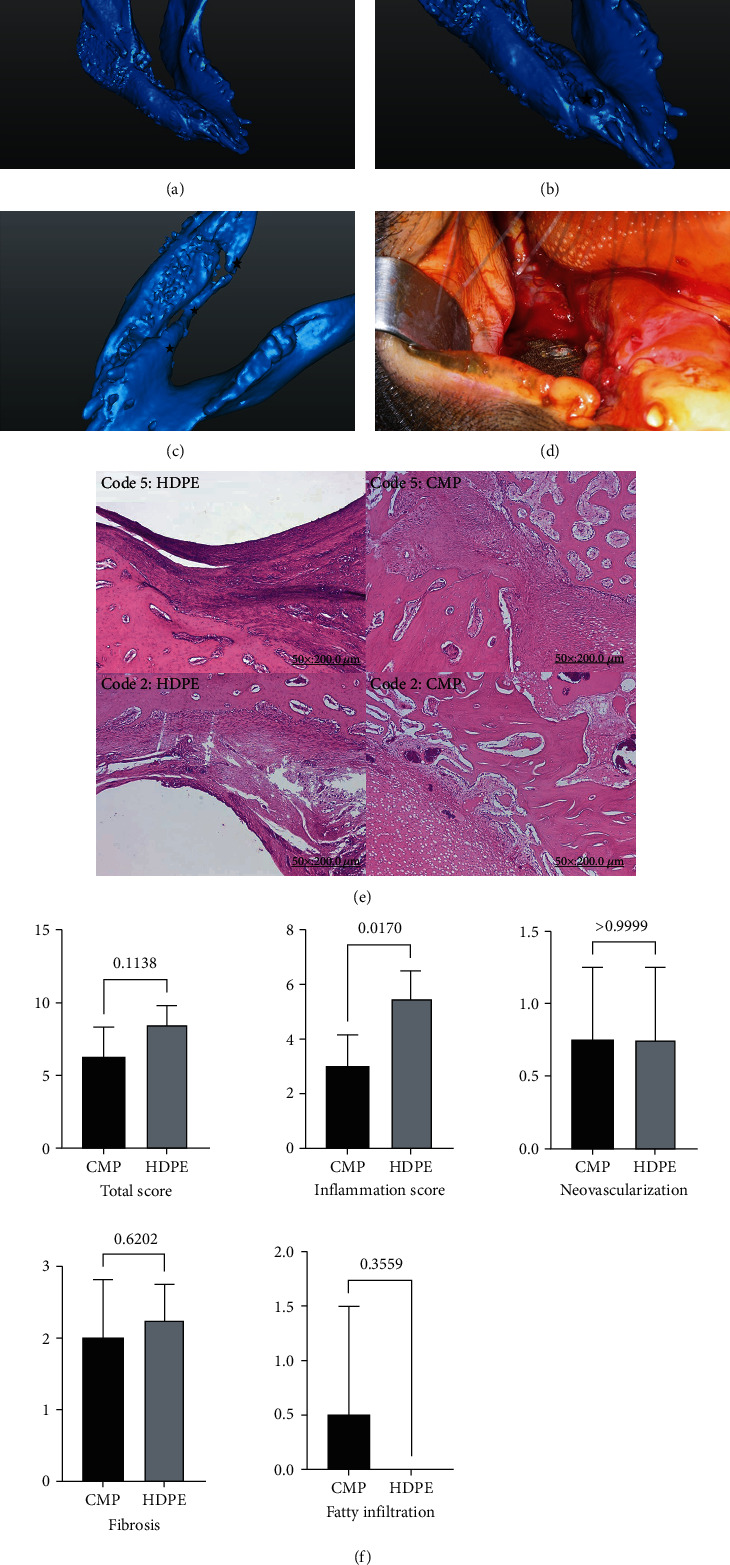
(a) Animal 3: according to radiological findings, the CMP remained in the original position. (b) The growth of the canine teeth loosened some of the screws in the front wing (stars). (c) The lingual cortex of the mandible crawled along the surface of the CMP, marked using stars. (d) CMP exposure in the oral cavity without inflammation, hemorrhage, necrosis, or purulent drainage. (e) Histological appearance (H&E staining, ×50) for CMP and HDPE groups in Animals 2 and 5. (f) Bar graph with means and standard deviations of histological analysis findings based on ISO 10993-6 scoring between the CMP and HDPE groups for the assessment of inflammatory status (*t* test). CMP was significantly less reactive than HDPE (*p* = 0.0170).

The progress of all animals after surgery is recorded in Table 8. We terminated Code 1 animal earlier, because we mistakenly selected an oversized drill, which caused the screws in the front wing of the CMP to fail to lock in tightly. In the second month, it was obvious that the CMP was loose and the front end of the CMP penetrated the animal's skin, so we decided to stop the experiment at this time point (Figure 7(b)). The rest of the animals completed the experiment, and there was no penetration of the skin of CMP. Code 3 and 5 animals had CMP exposure in the oral cavity (Figure 11(d)), but there were no significant signs of inflammation, hemorrhage, necrosis, and purulent drainage. No foreign body reaction was found, all wounds healed well, and we think this is because the material we use is a human compatible titanium alloy. All animals had normal eating conditions, and no significant weight loss or cachexia occurred within 3 months after operation.

#### 3.5.2. Radiological Results

The radiological evaluation focused on the localization of the CMP with regard to the mandibular defect. The computer tomography (CT) scans of the animals were taken after two months post operation except Code 1 animals. We found that a complete fracture line was observed over the connection between the CMP and rear wing in Code 4 animals and anterior segment had moved downward (Figure 7(e)). Nevertheless, some of the posterior stump of the anterior segment was still stuck in the rear mandible. We also found that the bones at both cutting ends crawled toward the middle parts and thought that CMP possibly played as a space maintainer.

In Code 3 animals, we found that the lingual cortex of the mandible crawled along the surface of the CMP which were marked by the star symbols in Figure 11(c). By the second month, it nearly occupied half of the space, and in the third month, there was even a bony front-to-back connection. In fact, there were similar findings in other animals.

Reviewing back to the animals, we selected 4-month-old pigs, so the permanent teeth were not completely erupted. As shown in Figure 11(b), the growth of the canine teeth caused some of the screws in the front wing of the CMP to be pushed away or even loosened, and this phenomenon should be considered in future experiments.

#### 3.5.3. Gross Examination of the Explanted Mandibles

The details of the macroscopic findings are presented in Table 8. Three months later, we performed sacrifice operations on the remaining four animals and observed the condition of the CMPs. Except in Code 4 animals, we found that although the remaining CMPs are still attached to the original position, they were more or less loose. We found that one to two of the 3 screws on the front wing were more likely to loosen than the screws on the rear wing (zero to one of the 6 screws). We observed that the rapid growth of lingual cortical bones and the eruption of canine teeth were the main two reasons for the loosening of the CMPs (Figures 6(b) and 6(c)).

#### 3.5.4. Histological Evaluation of Local Effects

The International Standard ISO-10993-6 [67] for biological evaluation of medical devices was employed for the assessment of the local effects after the implantation of the different biomaterials in this study. Detailed indexes and its scoring system used for biological evaluation of implanted materials were taken into account for inflammation, neovascularization, fibrosis, and fatty infiltration. The International Standard Classification of the biomaterials are attributed by the results obtained through the scoring system: the average subtotal inflammation score is multiplied by 2 and added to the average neovascularization, fibrosis, and fatty infiltration subtotal score. The local effects were evaluated by a comparison of the tissue response caused by the CMP and the controls (HDPE) and can be considered nonirritant (<2.9), slight (3.0-8.9), moderate (9.0–15.0), and severe (>15.0) depending on the score obtained (semiquantitative analysis).

The individual histopathological evaluations are presented in Table 9. The histological appearance from the CMP and HDPE groups for the assessment of inflammatory status is shown in Figure 11(e), and no foreign body reaction was found. In Figure 11(f), the histological analysis and ISO 10993-6 scoring proved that the CMP (score of 6.3 points) is less reactive as an implant compared to the HDPE group (score of 8.5 points) without significance (*p* = 0.1138) in total score. The inflammatory scoring proved that the CMP is less reactive compared to the HDPE group with significant difference (*p* = 0.0170). However, there was no significant difference between CMP and HDPE in neovascularization, fibrosis, and fatty infiltration score. Overall, the CMP and HDPE groups were considered slightly irritant.

## 4. Discussion

Compared with traditional reconstruction surgery, free microvascular flap reconstruction has more flexible, best postoperative aesthetics, highest patient satisfaction advantages, and fewer complications in the donor area. However, its disadvantages are the high complexity of the operation, the need to use an expensive operating microscope to perform the microvascular anastomosis, the long operation time, and the serious complications of the total or partial flap necrosis if vascular embolism occurs [29, 68–72]. Because the mandible is an entity with curvature, it is difficult to rebuild its original form clinically. Although the transplanted fibula can be designed for multisegmental osteotomy, it still cannot restore the original appearance. In addition, the height of the fibula itself is limited; although it can be folded in a manner to increase its height, the difficulty of the operation will increase correspondingly. New technologies for 3D-design using image processing and CAD techniques and manufacturing using metal 3D-printing methods offer advances in reconstructive surgery [6, 73]. Because the mandible must withstand forces from different directions to provide proper chewing function, the function of pressure dispersion must be considered except shape mimicking [29]. Hence, for reconstruction of severe mandible defects with customized implant, careful attention should be given to the stress shielding effect and the design of the front and rear wing connected to the resting bone. In our FEA experiment, we found that the use of our pressure-depressing structure can indeed disperse the stress. Nevertheless, in our mechanical fracture analysis, we found an incomplete fracture line over the posterior dummy mandible (Figure 4(a)). We made a modification to increase the lingual extension of the rear wing to disperse the stress, and the fatigue analysis was carried out with this modification without fracture observations.

FEA is a method of discrete analysis, which is different from other numerical methods based on mathematical discretization of boundary problem equations. FEA is a physical discretization method based on the domain under consideration, these finite elements have defined dimensions, physical properties, and simple geometric shapes, and they can simulate the behavior of complex physical systems.

In this experiment, we designed the PRD of CMP and used FEA to perform mechanical simulation analysis under four clinical situations. In this analysis, the thickness of the parallel hollow structures of the PRD increased to 0.5 mm (the original design is 0.3 mm) to amplify the analysis effect to facilitate our observation. According to the results of this analysis, although the total deformation caused by P1 was the smallest, the distribution of stress was not significantly improved. Although the displacement of P4 was the largest, it could properly adjust the stress distribution and equivalent strain in protrusion and right excursion conditions. Nevertheless, P3 had a good performance in the equivalent strain analysis in clenching and right excursion conditions, but there was no similar finding in the analysis of stress distribution. Therefore, according to the results of this analysis, we believed that P4 might be a relatively better PRD deployment among these four types of CMPs.

Titanium-made plates and screws are often used in the reconstruction of facial bones and mandibles. However, the stiffness of titanium is higher than that of the mandibles; this stiffness difference may cause the so-called stress shielding, which may cause bone resorption at stress concentrations in the long term [74–76]. This phenomenon is considered an adversary effect from the fixation plate. In some cases, the fixation device may have fatigue fracture if it continues to carry the majority of the load. Fixation device failures due to either stress shielding or stress concentration often need second-revision surgeries, and the difficulty of the second operation will also increase from scar contraction [36, 77, 78]. Cheng et al. [28] suggested that PEKK, which has a strength similar to that of the mandible, is a better choice for reconstruction, but it is currently only in the stage of in vitro experiments.

Researchers [28, 43] proposed to use a weighted topology optimization method to design a patient-specific mandibular implant to achieve the purpose of pressure release and weight reduction, but we used a pressure-reducing device to achieve this effect. In this study, we further performed fracture and fatigue test in CMP with P4 design according to ISO 14801, the test result showed that the maximum static pressure withstand value was 189 N (Figure 4(b)), and the CMP also passed five million fatigue tests under the fatigue stress test of 10 N-100 N under repeated stress (Figure 4(d)). The dummy mandible has no falling off or displacement, which is in line with the worst clinical situation. However, we found an incomplete crack line on the lingual side of the rear mandible (black triangle, Figure 4(a)). To solve this phenomenon that might be encountered clinically, we have extended a covered support structure on the lingual side of the rear wing to reduce the risk of fracture caused by lateral stress, and it also passed the fatigue test (Figure 4(c)).

Based on Perren's theory [79], adequate strain has an effect on the tissue differentiation during the fracture healing process. With strain values up to 2%, direct bone healing with lamellar bone formation occurs. Strain values between 2 and 10% induce callus formation and are tolerated by the three-dimensional configuration of newly forming bone tissue. However, when the strain reaches higher values than 10%, bone resorption prevails, and bone bridging does not occur [79]. Therefore, in the future, we should conduct experiments on different pressure relief structures to tell us more information.

In FEA, we did not consider interfacial protruded structures that enhance osseointegration and friction at the interface between the implant and the bone interface. Suitable porosity of interfaces can be considered in titanium implant/bone interfaces during the 3D manufacture process, and we contained this design in CMP in our animal experiment, despite the influence of pore sizes of implant materials on bone in-growth that remains controversial and could result in decreases in stiffness and strength [80–82]. Back to our animal experiments, we did not consider well that the outer layer of the mandible contains thick cortical bone causing us to apply a squeezing force to close the gap while placing CMP. Although this squeezing force might allow protrusions to set deeper into the internal structure of the bone, just like the function of a locking screw system which prevent screw toggle, and increase plate resistance to axial loads compared with conventional screws [83], it might also cause microcrack in the cortical bone.

There are still some limitations in our simulations. First, the experiments were performed by using pigs with an average age of 4 months, and the animals were sacrificed after 3 months. Although we choose fast-growing young pigs, 3 months does not ensure mature osteointegration which was set up in FEA. Second, the material properties of bone are simplified to be linear and isotropic. However, the material properties of bone are closer to be anisotropic and inhomogeneous; therefore, those conditions can be adopted in the future study to bring the results closer to the real clinical situation. Third, in an in vivo study, a complete fracture line was observed over the connection between the CMP and rear wing in Code 4 animals (Figure 7(e)). We found that most of the maximum stress was concentrated at the junction of the main body and the wings (508.53 MPa in P4) by animal FEA. These findings seem to have similar results, but more experiments are needed to verify in the future. Last, in our research, maximum principal stress criteria are used in bone stress analysis. However, the Hills criterion—which is an extension of the von Mises criterion—was used by Sharma et al. [84] for the cortical bone, while the Tsai-Wu criterion, which was originally expressed for composite materials, has been used by Keaveny et al. [85] to predict the multiaxial failure of the trabecular bone. If these properties and criterions can be added to our experiments in the future, our models may be closer to reality.

The progress of all animals after surgery was recorded, and we terminated Code 1 animals earlier. The wrong choice of drill in operation may be the main reason for screw loosening and subsequent CMP displacement (Figure 7(b)). Code 3 and 5 animals had CMP exposure in the oral cavity, but there were no significant signs of inflammation and foreign body reaction. We think this is because titanium is considered a well-known biocompatible metal after long-term verification [86]. In the future, the intraoral surface of CMP must ensure sufficient soft tissue coverage using regional or microvascular flap to increase its seal capacity.

We found that a complete fracture line was observed over the connection between the CMP and rear wing in Code 4 animals (Figure 7(e)), and this might be caused by a weak connection design of CMP. On the other hand, higher eating frequency, continuous rubbing of the lateral face against the railing of the residence due to skin paresthesia post operation, and being unable to cooperate and obey must also be considered.

Pigs have a similar bone regeneration rate to humans [87, 88], and dentition is replaced with permanent teeth by the time they are 20 months old [89]. Reviewing back to the animals, we selected 4-month-old pigs, so the permanent teeth were not completely erupted and were in a period of rapid bone and weight growth [89]. Figure 11(b), and postoperative specimens showed that one to two of the 3 screws on the front wing were more likely to loosen. We concluded that the rapid growth of lingual cortical bones and the eruption of canine teeth might be the main two reasons for the loosening of the CMPs. Therefore, in future experiments, the development of animals must also be considered.

Titanium is considered the most biocompatible metal due to its resistance to corrosion from bodily fluids, bioinertness, capacity for osseointegration, and high fatigue limit [86]. Histological appearance from the CMP and HDPE groups for the assessment of inflammatory status was shown, and no foreign body reaction was found. ISO 10993-6 scoring showed that CMP is less reactive compared to HDPE with significant difference (*p* = 0.0170).

The current surgical success rate of free fibular graft for mandibular reconstruction is reported from 92% to 96%, and the dental implant survival rate on fibula is 79.9%–91%. However, the final prosthetic rehabilitation success rate is only 42.9% [90]. In addition to the lack of bone volume, height, and width, its inverted triangle shape is not conducive to subsequent dental implant placement and denture rehabilitation. CMP-integrated dental implants are believed to be the future development trend, but more research is needed for verification.

## 5. Conclusion

In this research, we designed PRD for CMP and conducted a FEA experiment. We found that putting PRD on the back end of the CMP body is conducive to the dispersion of stress. We also performed fracture test and fatigue test on CMP with P4 design and made modification on rear lingual extension. In the animal experiments, the inflammatory scoring proved that CMP is less reactive compared to the HDPE group with significant difference, part of retention screws on the front wing were partially loosened, and that might be induced from the pushing effect by the germination of the canine and permanent teeth. In the future, customized mandibular implants combined with dental implants are the future trend, but more research to verify its biocompatibility and efficacy is still necessary.

## Figures and Tables

**Table 1:** Number of nodes and elements for each mesh in human and animal models.

	Human models				Animal models			
	P1	P2	P3	P4	P1	P2	P3	P4
Mesh nodes	136067	147084	141606	142018	83112	85570	84318	84252
Mesh elements	75548	81152	78422	78655	46306	47185	46730	46688

**Table 2:** Material properties of different parts in the finite element model.

Type of material	Young's modulus (MPa)	Poisson's ratio
Mandible	8700	0.28
Ti-6Al-4V (CMP)	105000	0.3
Ti-6Al-4V (screws)	105000	0.3

**(a):** 

	Clenching	Resting muscle weight (N)	Unit vector coordinates			Protrusion	Stretched muscle weight (N)	Unit vector coordinates		
	Total force		*X*	*Y*	*Z*	Total force		*X*	*Y*	*Z*
L sup. masseter	1.00	190.40	32.68	-93.65	201.61	1.06	201.61	34.27	-85.70	179.24
L deep masseter	1.00	81.60	25.23	-13.49	87.17	1.07	87.17	26.00	-8.16	82.80
R ant. temporalis	1.00	158.00	-10.63	4.58	169.55	1.07	169.55	-10.86	14.80	168.55
R mid. temporalis	1.00	95.60	-28.83	44.52	103.04	1.08	103.04	-29.11	50.83	84.77
R post. temporalis	1.00	75.60	-23.88	62.60	81.64	1.08	81.64	-23.98	67.70	38.81
L ant. temporalis	1.00	158.00	-3.19	6.76	169.66	1.07	169.66	-3.25	17.19	168.75
L mid. temporalis	1.00	95.60	18.10	48.97	103.18	1.08	103.18	18.20	55.32	85.17
L post. temporalis	1.00	75.60	14.29	65.11	81.71	1.08	81.71	14.31	70.11	39.46
L med. pterygoid	1.00	174.80	-83.94	-60.27	185.81	1.06	185.81	-87.35	-48.70	156.60
R lat. pterygoid	1.00	66.90	46.58	-32.02	68.71	1.03	68.71	51.83	-29.58	-34.06
L lat. pterygoid	1.00	66.90	-44.59	-35.91	68.59	1.03	68.59	-49.72	-34.02	-32.78
R digastric	1.00	40.00	-2.55	31.29	43.00	1.07	43.00	-2.60	36.15	-23.14
L digastric	1.00	40.00	6.46	30.89	43.01	1.08	43.01	6.57	35.82	-22.88
L1-U1							100.00	-2.02	-69.02	-72.33
43-13			19.24	46.18	-141.41					
33-23			-23.62	-33.06	-144.39					
36-26			-23.27	23.27	-298.19					

**(b):** 

	Right excursion	Stretched muscle weight (N)	Unit vector coordinates			Left excursion	Stretched muscle weight (N)	Unit vector coordinates		
	Total force		*X*	*Y*	*Z*	Total force		*X*	*Y*	*Z*
L sup. masseter	1.04	197.71	30.35	-87.21	174.82	1.06	201.32	34.40	-100.04	171.28
L deep masseter	1.05	85.35	25.54	-7.09	81.13	1.06	86.21	26.05	-14.69	80.86
R ant. temporalis	1.07	169.26	-5.82	5.85	169.06	1.07	168.36	-17.19	23.29	165.85
R mid. temporalis	1.07	102.00	-26.78	46.67	86.66	1.08	103.31	-32.37	55.11	81.16
R post. temporalis	1.06	80.31	-22.38	65.80	40.22	1.08	82.00	-26.03	69.32	35.24
L ant. temporalis	1.06	166.86	1.45	26.91	164.67	1.06	167.55	-8.50	7.75	167.16
L mid. temporalis	1.08	102.91	21.10	60.98	80.17	1.06	101.15	15.91	51.84	85.39
L post. temporalis	1.08	81.99	16.18	72.75	34.16	1.06	79.91	12.50	68.85	38.59
L med. pterygoid	1.05	183.04	-96.28	-47.81	148.15	1.06	184.80	-88.27	-65.15	148.71
R lat. pterygoid	1.05	70.04	50.48	-33.93	-34.72	1.03	68.62	51.11	-23.56	-39.26
L lat. pterygoid	1.04	69.25	-49.14	-27.61	-40.24	1.05	70.46	-47.17	-37.49	-36.53
R digastric	1.06	42.28	-2.23	34.33	-24.58	1.07	42.72	-5.24	34.56	-24.55
L digastric	1.07	42.65	7.21	34.27	-24.34	1.06	42.26	4.04	33.97	-24.82
L1-U1										
43-13							150.00	107.85	-66.37	-80.39
33-23										
36-26		300.00	-92.34	-229.89	-169.19		300.00	180.66	-66.80	-230.00

**Table 4:** The calculated forces in *X*, *Y*, and *Z* directions in clenching position in the animal model.

	Clenching muscle weight (N)	Unit vector coordinates		
		*X*	*Y*	*Z*
L sup. masseter	304.4	23.95	-102.42	285.65
L deep masseter	188.8	36.57	-78.80	167.62
R ant. temporalis	64.8	4.68	10.02	63.85
R mid. temporalis	73.2	6.69	35.28	63.79
R post. temporalis	73.2	11.76	52.76	49.36
L ant. temporalis	64.8	-7.39	12.52	63.15
L mid. temporalis	73.2	-7.80	36.81	62.79
L post. temporalis	73.2	-13.91	53.26	48.25
L med. pterygoid	226.8	-123.62	-51.48	183.05
R lat. pterygoid	100.4	55.31	-50.57	-66.81
L lat. pterygoid	100.4	-53.38	-51.06	-67.99
R digastric	56.4	-0.18	-56.24	-4.26
L digastric	56.4	-1.16	-56.23	-4.28
Molar occlusion	250.00	-73.12	42.03	-235.34
Incisor occlusion	90.00	9.22	51.77	-73.04

**Table 5:** Number of elements, number of nodes, computational cost and absolute mean error (MAE), mean square error (MSE), and root mean square error (RMSE) corresponding to each of the standardized tests for FE models under different mesh sizes (1.5, 2, 2.5, 2.75, and 3 mm) and formulation (linear/quadratic).

	Mesh nodes	Mesh elements	Elapsed time (min)	MAE	MSE	RMSE
1.5 mm/linear	69130	247200	18.53	90.30	9680.69	98.39
1.5 mm/quadratic	430098	244033	32.53	58.96	3986.32	63.14
2 mm/linear	53926	193317	13.35	91.35	9927.45	99.64
2 mm/quadratic	332289	189147	17.33	58.89	3977.68	63.07
2.5 mm/linear	40929	143764	3.97	92.78	10310.08	101.54
2.5 mm/quadratic	245998	138330	17.02	59.64	4086.01	63.92
2.75 mm/linear	40789	143158	11.02	94.38	10671.66	103.30
2.75 mm/quadratic	244862	137616	18.77	**59.67**	**4090.02**	**63.95**
3 mm/linear	40759	142967	10.62	94.06	10609.93	103.00
3 mm/quadratic	244923	137677	16.73	58.84	3971.40	63.02

**Table 6:** Maximum values for total deformation, von Mises stress, von Mises equivalent strain, and principal stress of CMP-PRD and retentive screws by FEA under the clenching (CP1 to CP4), protrusion (PP1 to PP4), and right (RP1 to RP4) and left excursion conditions (LP1 to LP4) in the human model.

Human model	Total deformation max (mm)	Equivalent stress max (MPa)	Equivalent elastic strain max (mm/mm)	Strain energy max (mJ)	Equivalent stress max-ant. screws (MPa)	Equivalent stress max-post. screws (MPa)	Principal stress max-ant. mandible (MPa)	Principal stress max-post. mandible (MPa)
CP1	1.252	1250.275	0.022	0.520	227.200	430.912	56.589	91.526
CP2	1.871	1104.978	0.023	1.072	215.658	436.295	70.749	92.837
CP3	1.242	1198.720	0.023	0.541	219.410	459.747	54.929	95.803
CP4	1.751	1194.329	0.021	0.491	215.812	409.830	69.877	87.207
PP1	1.178	1323.087	0.022	0.546	115.568	420.086	49.051	87.805
PP2	1.310	1172.766	0.024	0.709	97.663	470.425	56.489	96.089
PP3	1.298	1270.596	0.023	0.702	98.762	465.359	56.551	94.826
PP4	1.341	1245.379	0.023	0.698	104.558	452.524	55.651	93.018
LP1	1.133	1319.820	0.022	0.511	132.779	459.036	52.796	95.807
LP2	0.955	1172.592	0.024	0.563	128.120	471.824	40.125	98.156
LP3	1.107	1264.902	0.024	0.467	129.189	483.623	48.622	98.668
LP4	0.914	1239.435	0.023	0.560	133.562	451.397	40.646	94.789
RP1	1.588	1369.503	0.021	0.915	111.398	409.992	71.491	88.334
RP2	1.959	1210.231	0.023	1.425	91.646	477.056	89.648	96.940
RP3	1.774	1308.170	0.023	1.233	92.033	463.897	83.644	95.229
RP4	1.991	1283.492	0.023	1.408	97.475	460.473	88.564	94.689

**Table 7:** Maximum values for total deformation, von Mises stress, von Mises equivalent strain, and principal stress of CMP-PRD and retentive screws by FEA under the clenching (P1 to P4) in the animal model.

Animal model	Total deformation max (mm)	Equivalent stress max (MPa)	Equivalent elastic strain max (mm/mm)	Strain energy max (mJ)	Equivalent stress max-ant. screws (MPa)	Equivalent stress max-post. screws (MPa)	Principal stress max-ant. mandible (MPa)	Principal stress max-post. mandible (MPa)
P1	0.501	516.599	0.009	0.617	73.232	52.619	59.038	37.335
P2	0.503	531.027	0.010	0.652	78.805	55.402	58.993	37.125
P3	0.501	484.110	0.010	0.599	87.966	46.309	59.039	37.413
P4	0.501	508.534	0.010	0.688	86.485	49.026	59.074	37.935

**Table 8:** Postoperative status and progress of all animals.

Animal code	Operation date	Sacrifice date	CMP position	Skin	Oral mucosa	Infection with pus discharge	Screw status (loss/all screws)	Completed experiment
1	2020/04/06	2020/06/15	Obvious displacement	Front wing skin penetration	Intact	None	Front: 3/3 Rear: 1/6	No (2^nd^ month)
2	2020/04/06	2020/07/22	Correct position	Intact	Intact	None	Front: 2/3 Rear: 0/6	Yes
3	2020/04/13	2020/07/22	Correct position	Intact	2x1 cm exposure	None	Front: 1/3 Rear: 2/6	Yes
4	2020/04/13	2020/07/22	Rear wing fracture	Intact	Intact	None	Front: 1/3 Rear: 0/6	Yes
5	2020/04/20	2020/07/22	Correct position	Intact	0.5 x 0.5 cm exposure	None	Front: 1/3 Rear: 1/6	Yes

**Table 9:** Comparison of histopathological results between CMP and HDPE.

Implantation interval	3 months							
Group	CMP site				Control HDPE			
Animal number	2393-03-R	2397-04-R	2395-01-R	2398-02-R	2393-03-L	2397-04-L	2395-01-L	2398-02-L
Inflammation	Score							
Polymorphonuclear	1	0	0	1	1	1	1	1
Lymphocytes	1	1	1	1	1	2	2	2
Plasma cells	0	0	0	0	0	0	0	0
Macrophages	0	0	0	0	0	1	0	0
Giant cells	0	0	0	0	0	0	0	0
Necrosis	0	0	0	0	0	0	0	0
Subtotal (×2)	4	2	2	4	4	6	6	6
Neovascularization	1	1	0	1	1	1	0	1
Fibrosis	3	2	2	1	2	3	2	2
Fatty infiltration	0	0	0	2	0	0	0	0
Subtotal	4	3	2	4	3	4	2	3
Total	8	5	4	8	7	10	8	9
Group total^b^	25				34			
Average^a^	6.3				8.5			
Traumatic necrosis	0	0	0	0	0	0	0	0
Foreign body debris	0	0	0	0	0	0	0	0
No. of sites examined	1	1	1	1	1	1	1	1

^a^Average is the sum of scores among groups/numbers of recognizable implantation sites. Used to determine irritation ranking, which is shown below and served as the conclusion. A negative difference was coded as 0. ^b^Group total is the sum of scores among groups. Rating score is minimal or no reaction: 0.0–2.9; slight reaction: 3.0–8.9; moderate reaction: 9.0–15.0; and severe reaction: ≥15.1.

## Data Availability

We prefer authors to deposit their data in a public repository that meets appropriate standards of archiving, citation and curation (see below). Having data available in a structured database carries significant benefits. Such repositories typically allow the data to be stored in native formats, which maximizes the potential for analysis, reuse, and verification. This also makes it easier for users to search, filter, and analyze the data. Importantly, such repositories conform to a minimum set of best practices and provide appropriate standards of curation (e.g., a common structure and collection of metadata).

## Supplementary Materials

Supplementary Materials Table S1 and Figure S1 showed the results of mesh convergence study in the human model. Table S2 and Figure S2 showed the results of mesh convergence study in the animal model. Figures S3A-3H showed higher resolution from Figure 3 in the main text to facilitate numerical interpretation. Figures S4A-4C showed higher resolution from Figure 10 in the main text to facilitate numerical interpretation. Figures S5A-5B showed local magnification of the two groups with linear and quadratic formulation from Figure 5(e) in the main text.
