# Supplementary material for: The Use of Customized Three-Dimensionally Printed Mandible Prostheses with a Pressure-Reducing Device: A Finite Element Analysis in Different Chewing Positions, Biomechanical Testing, and In Vivo Animal Study Using Lanyu Pigs
Source: Biomed Res Int. 2022 Mar 16;2022:9880454. doi: 10.1155/2022/9880454 (PMC8942632; doi:10.1155/2022/9880454)
Supplement: Supplementary Materials — Table S1 and Figure S1 showed the results of mesh convergence study in the human model. Table S2 and Figure S2 showed the results of mesh convergence study in the animal model. Figures S3A-3H showed higher resolution from Figure 3 in the main text to facilitate numerical interpretation. Figures S4A-4C showed higher resolution from Figure 10 in the main text to facilitate numerical interpretation. Figures S5A-5B showed local magnification of the two groups with linear and quadratic formulation from Figure 5(e) in the main text. [file 9880454.f1.docx]

| Mesh Elements | Mesh Nodes | Equivalent Stress Maximum (MPa) | Change (%) | Equivalent Stress -front wing Maximum (MPa) | Change (%) | Equivalent Stress -rear wing Maximum (MPa) | Change (%) | Equivalent Stress -ant screws Maximum (MPa) | Change (%) | Equivalent Stress -post screws Maximum (MPa) | Change (%) |
| --- | --- | --- | --- | --- | --- | --- | --- | --- | --- | --- | --- |
| 44092 | 79203 | 824.355 |  | 70.994 |  | 824.355 |  | 59.835 |  | 153.837 |  |
| 46911 | 84098 | 912.480 | 10.690 | 73.519 | 3.558 | 912.480 | 10.690 | 79.570 | 32.983 | 180.328 | 17.220 |
| 49833 | 91110 | 995.697 | 9.120 | 75.788 | 3.086 | 995.697 | 9.120 | 91.488 | 14.978 | 215.744 | 19.640 |
| 52669 | 96653 | 1053.645 | 5.820 | 78.286 | 3.296 | 1053.645 | 5.820 | 98.211 | 7.348 | 282.342 | 30.869 |
| 59776 | 108993 | 1175.814 | 11.595 | 81.577 | 4.203 | 1175.814 | 11.595 | 92.962 | -5.345 | 250.115 | -11.414 |
| 68670 | 124066 | 1178.088 | 0.193 | 82.240 | 0.813 | 1178.088 | 0.193 | 93.710 | 0.804 | 256.105 | 2.395 |
| 75548 | 136067 | 1307.367 | 10.974 | 87.618 | 6.539 | 1307.367 | 10.974 | 115.062 | 22.785 | 405.829 | 58.462 |
| 80475 | 144779 | 1308.969 | 0.123 | 87.744 | 0.145 | 1308.969 | 0.123 | 113.682 | -1.199 | 407.867 | 0.502 |
| 94349 | 169146 | 1313.921 | 0.378 | 87.881 | 0.156 | 1313.921 | 0.378 | 114.881 | 1.055 | 390.580 | -4.238 |

Table S1. Results of mesh convergence study in human model.


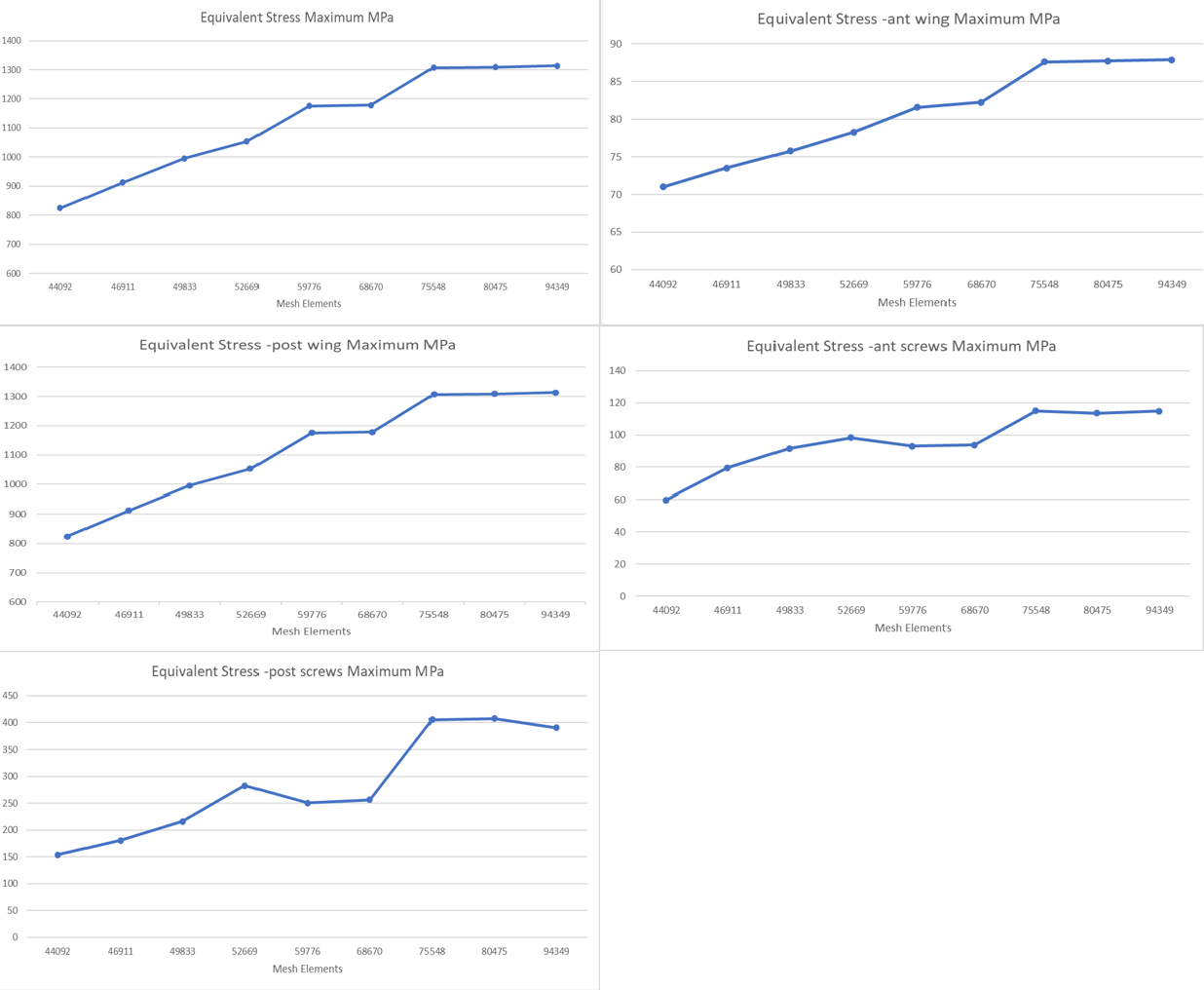


Figure S1. Results of mesh convergence study in human model.

| Mesh Elements | Mesh Nodes | Equivalent Stress Maximum (MPa) | Change (%) | Front wing Maximum (MPa) | Change (%) | Rear wing Maximum (MPa) | Change (%) | Equivalent Stress -ant screws Maximum (MPa) | Change (%) | Equivalent Stress -post screws Maximum (MPa) | Change (%) |
| --- | --- | --- | --- | --- | --- | --- | --- | --- | --- | --- | --- |
| 34594 | 63263 | 469.362 |  | 128.512 |  | 148.311 |  | 36.889 |  | 22.210 |  |
| 36704 | 66736 | 505.208 | 7.637 | 134.606 | 4.742 | 153.040 | 3.188 | 38.868 | 5.364 | 36.494 | 64.317 |
| 37790 | 68555 | 503.880 | 0.377 | 141.387 | 5.038 | 153.001 | -0.025 | 45.328 | 16.621 | 35.995 | -1.368 |
| 39246 | 71323 | 507.114 | 1.873 | 140.927 | -0.326 | 153.815 | 0.532 | 55.327 | 22.058 | 41.017 | 13.950 |
| 40535 | 74276 | 513.316 | 3.237 | 141.559 | 0.449 | 154.082 | 0.174 | 58.305 | 5.384 | 50.891 | 24.073 |
| 43037 | 78549 | 523.528 | 6.594 | 140.221 | -0.945 | 156.047 | 1.275 | 57.894 | -0.705 | 50.305 | -1.151 |
| 56409 | 101749 | 547.162 | 5.870 | 143.017 | 1.994 | 158.544 | 1.600 | 58.450 | 0.960 | 49.400 | -1.798 |
| 61483 | 110336 | 554.258 | -0.285 | 143.247 | 0.161 | 159.004 | 0.291 | 58.707 | 0.440 | 49.362 | -0.077 |

Table S2. Results of mesh convergence study in animal model.


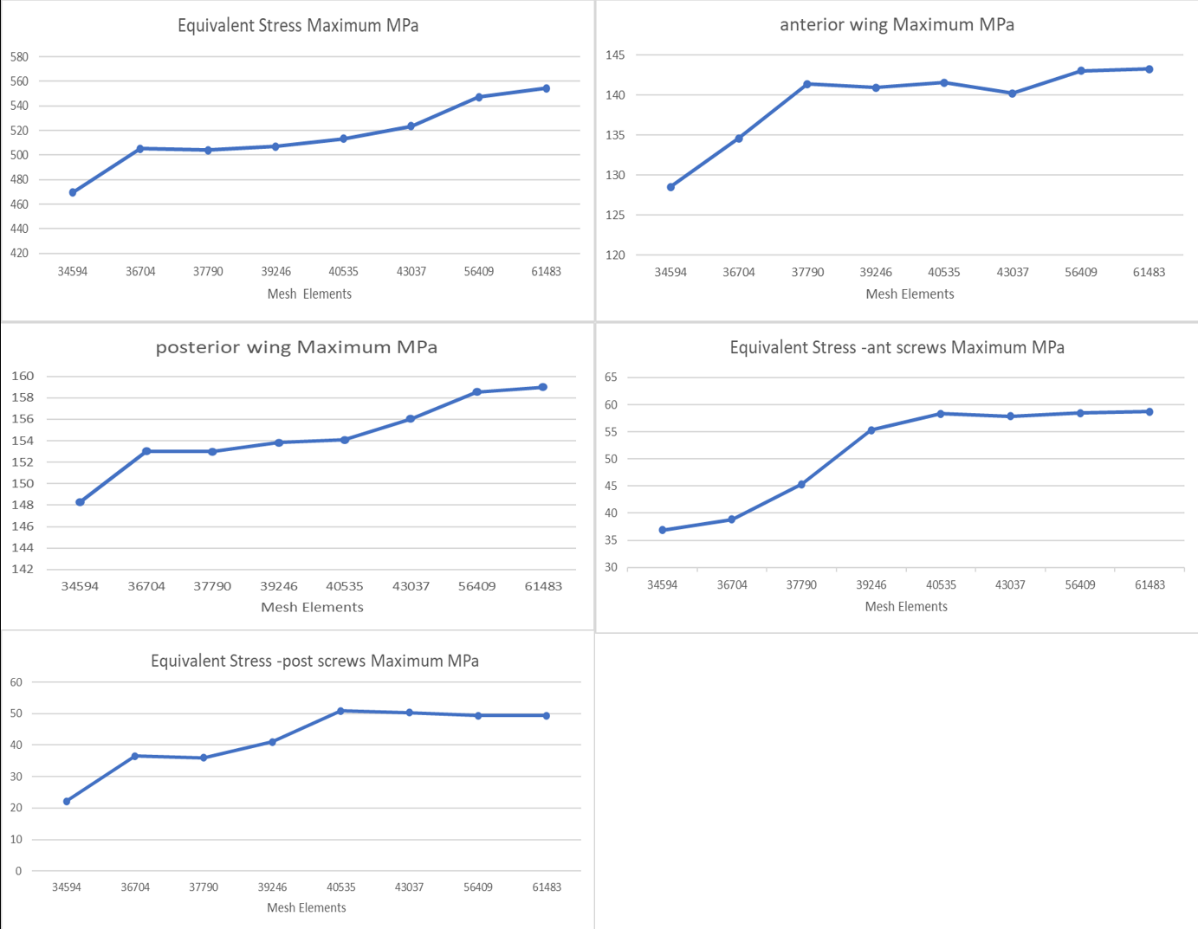


Figure S2. Results of mesh convergence study in animal model.


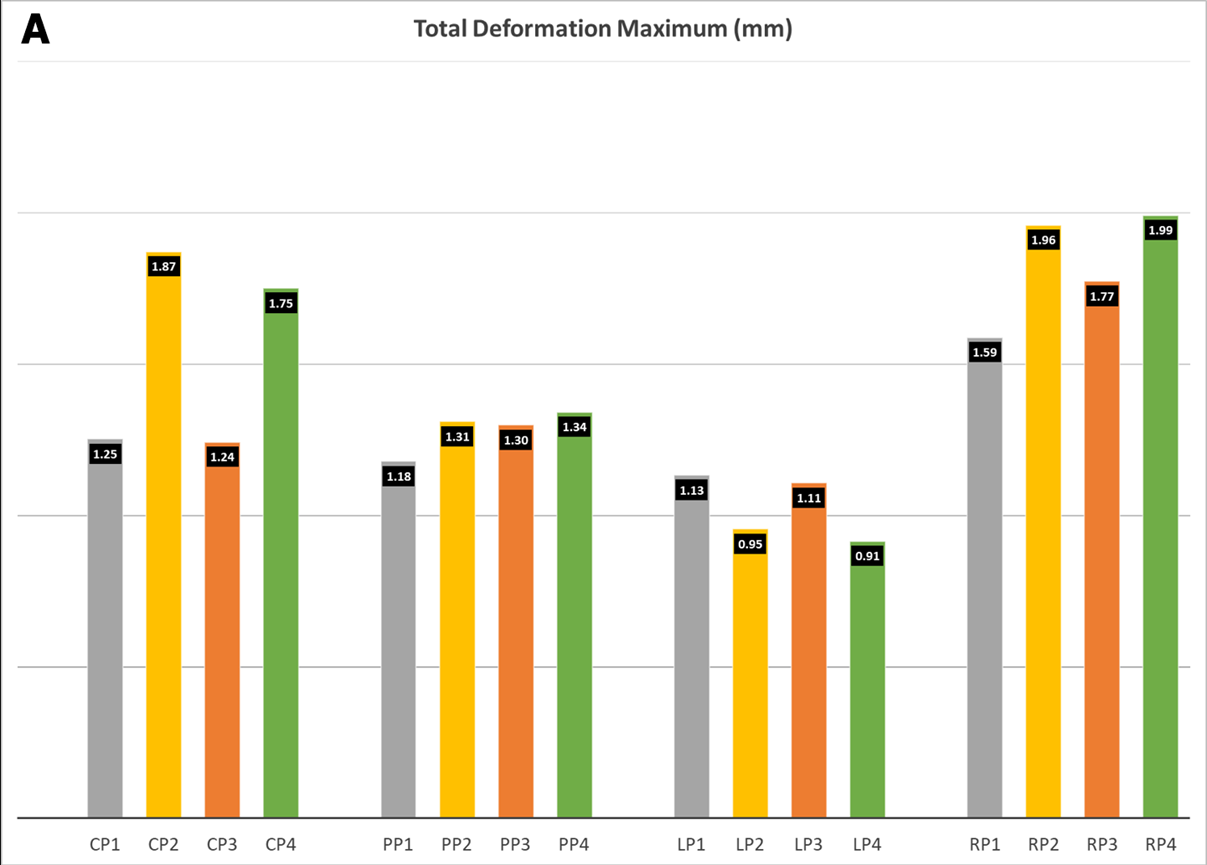


Figure S3A. Level of total deformation of whole components, CMP-PRD (P1, P2, P3, and P4) by FEA under the clenching (CP1-CP4), protrusion (PP1 to PP4), and left (LP1 to LP4) and right excursion (RP1 to RP4) conditions.


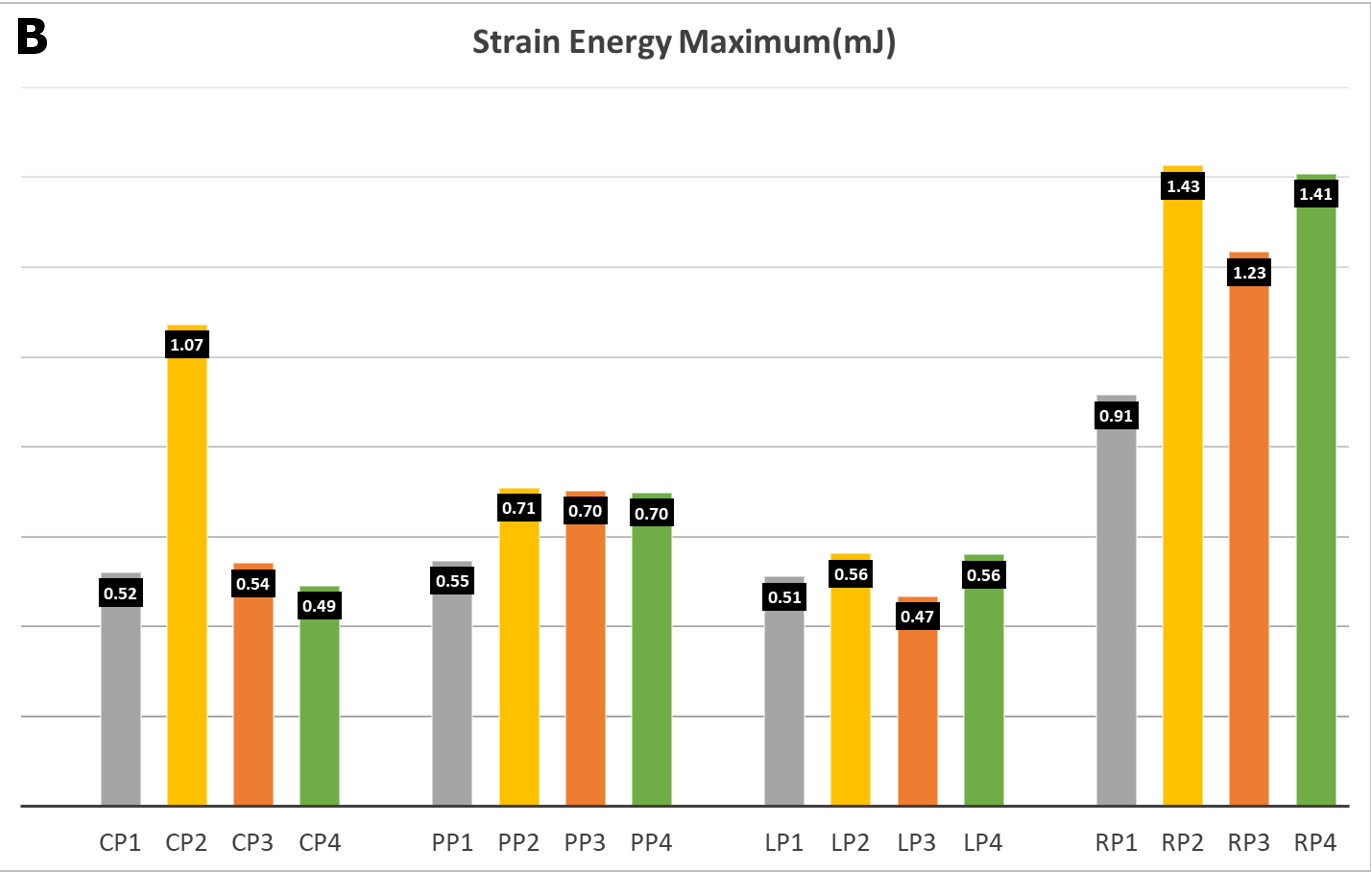


Figure S3B. Level of strain energy of whole components, CMP-PRD (P1, P2, P3, and P4) by FEA under the clenching (CP1-CP4), protrusion (PP1 to PP4), and left (LP1 to LP4) and right excursion (RP1 to RP4) conditions.


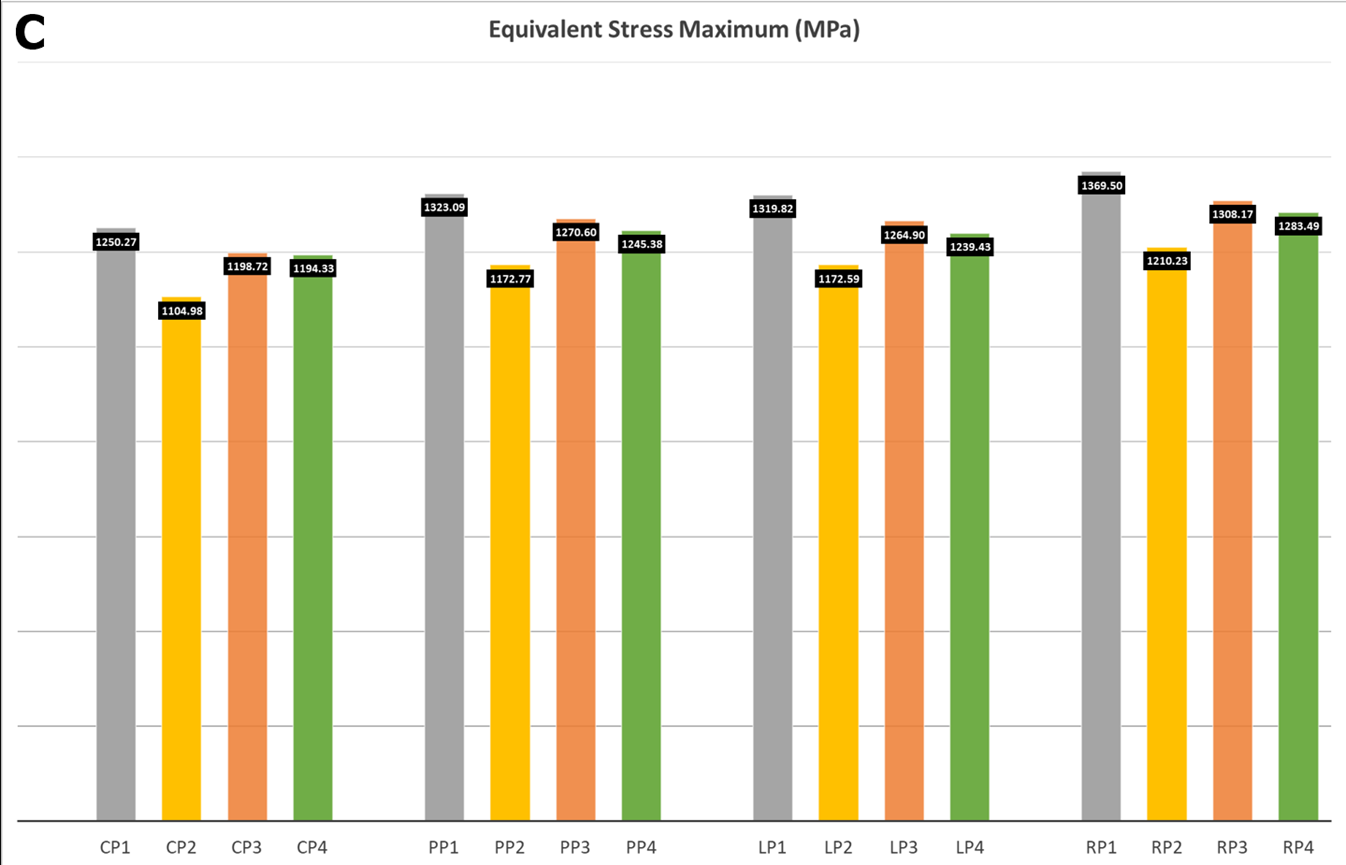


Figure S3C. Level of von Mises stress of whole components, CMP-PRD (P1, P2, P3, and P4) by FEA under the clenching (CP1-CP4), protrusion (PP1 to PP4), and left (LP1 to LP4) and right excursion (RP1 to RP4) conditions.


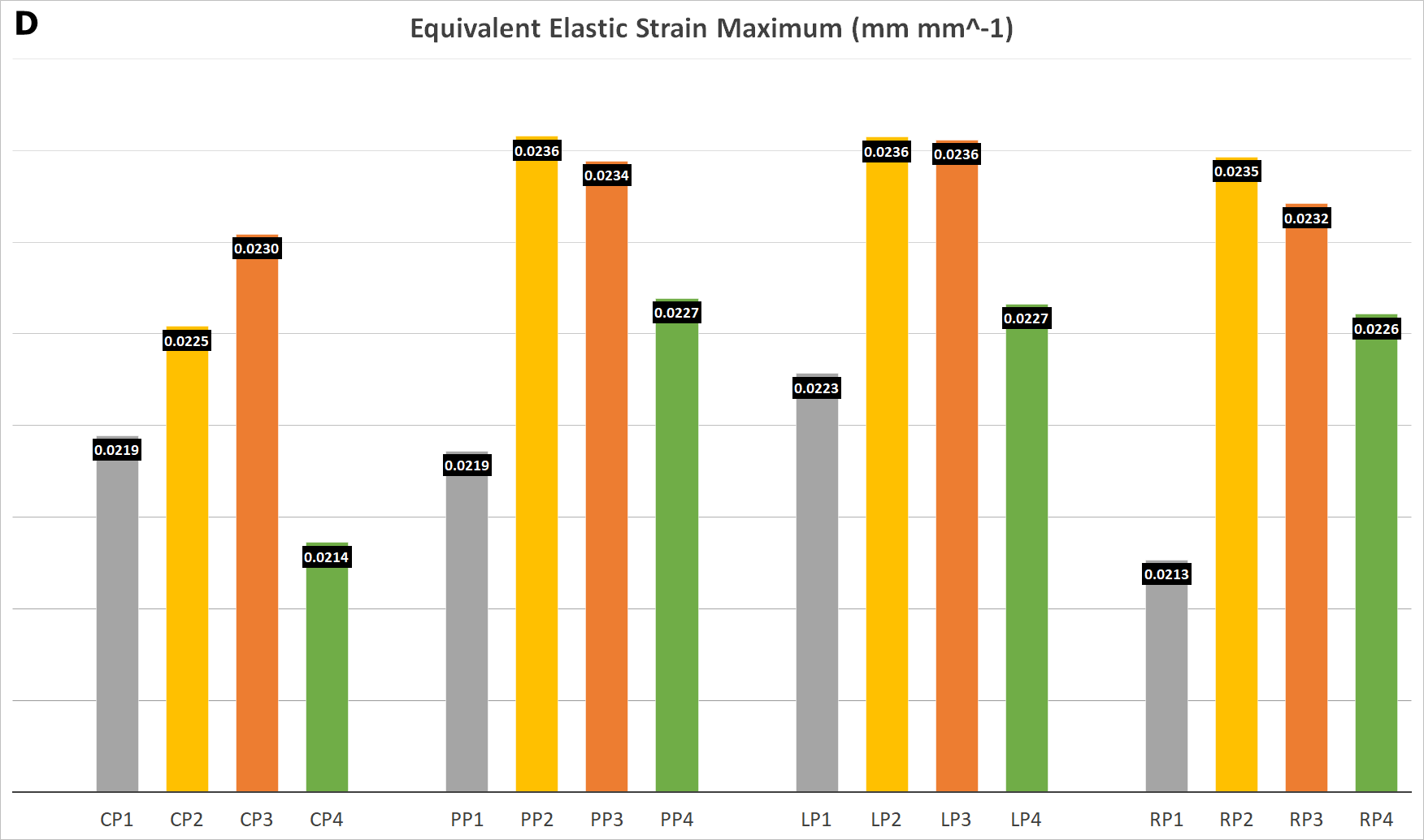


Figure S3D. Level of Von Mises equivalent strain of whole components, CMP-PRD (P1, P2, P3, and P4) by FEA under the clenching (CP1-CP4), protrusion (PP1 to PP4), and left (LP1 to LP4) and right excursion (RP1 to RP4) conditions.


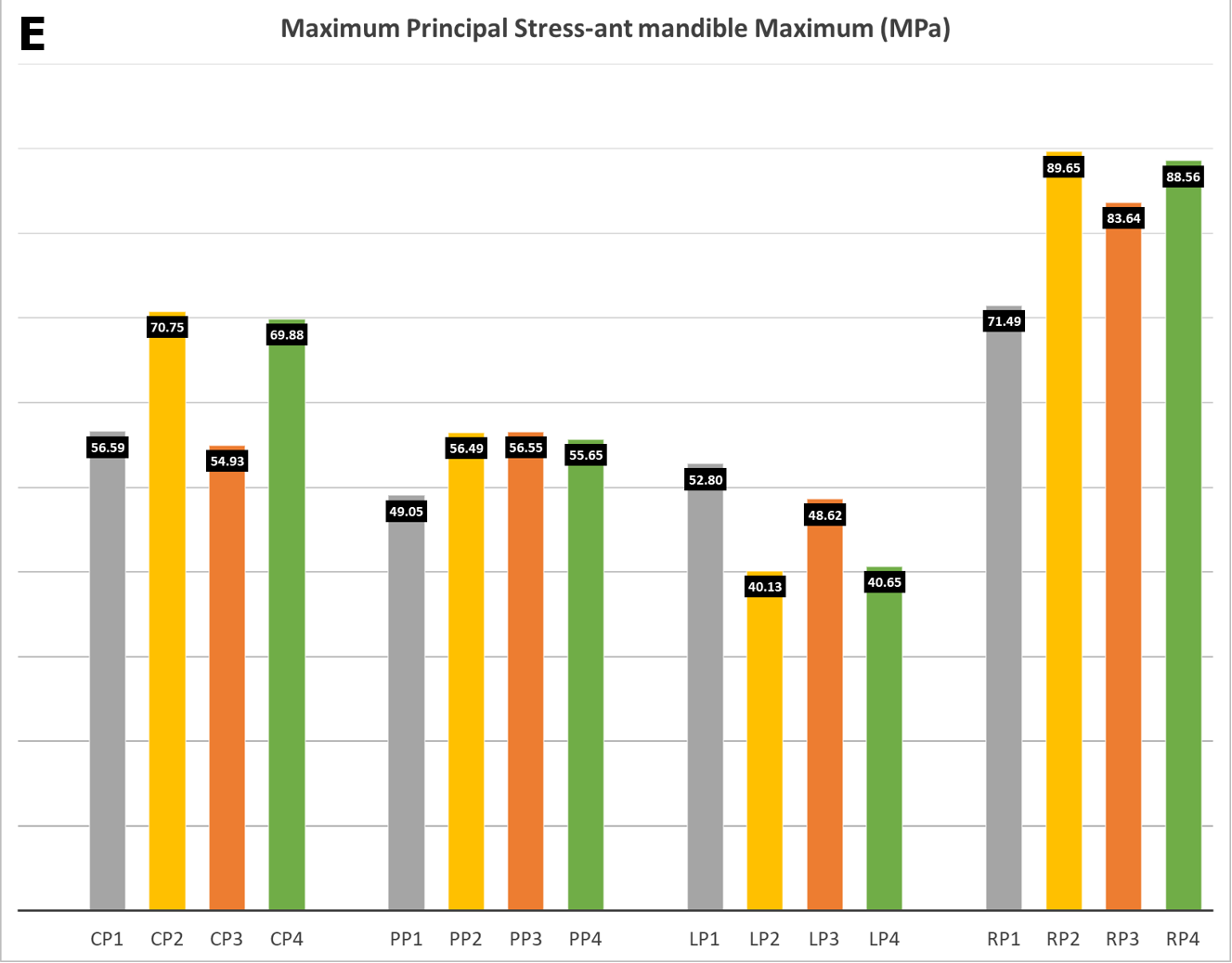


Figure S3E. Level of maximum principal stress of anterior mandible component by FEA under the clenching (CP1-CP4), protrusion (PP1 to PP4), and left (LP1 to LP4) and right excursion (RP1 to RP4) conditions.


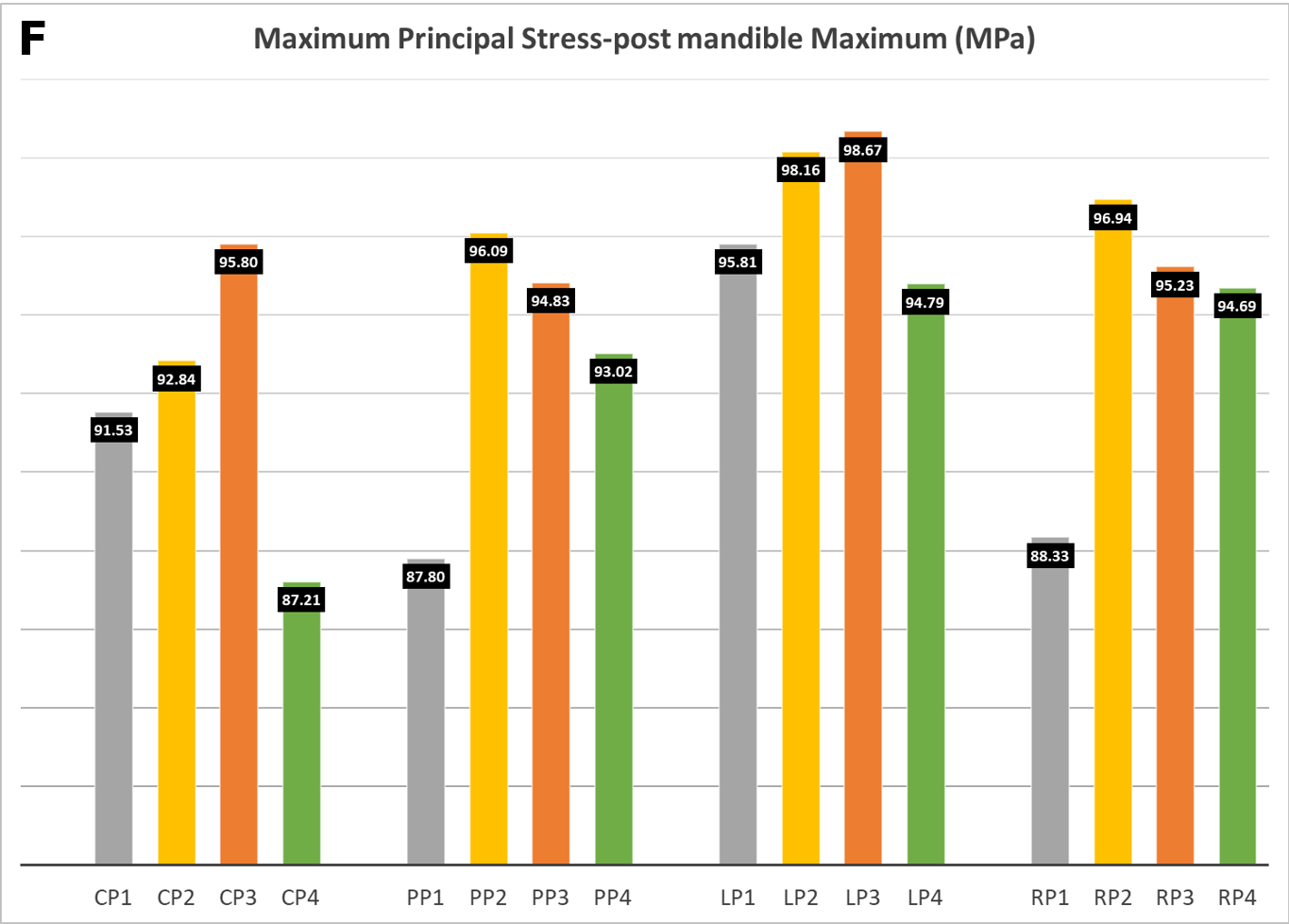


Figure S3F. Level of maximum principal stress of posterior mandible component by FEA under the clenching (CP1-CP4), protrusion (PP1 to PP4), and left (LP1 to LP4) and right excursion (RP1 to RP4) conditions.
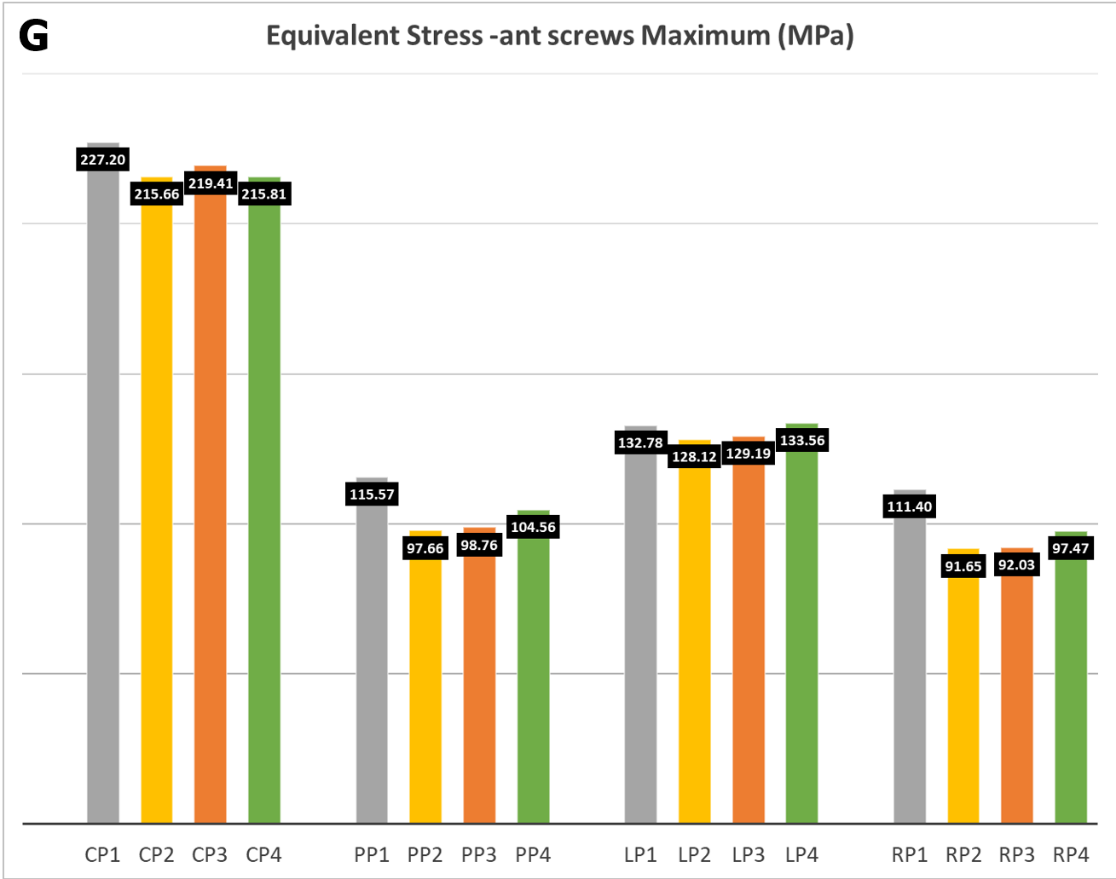


Figure S3G. Level of von Mises equivalent stress of anterior screws by FEA under the clenching (CP1-CP4), protrusion (PP1 to PP4), and left (LP1 to LP4) and right excursion (RP1 to RP4) conditions. conditions.
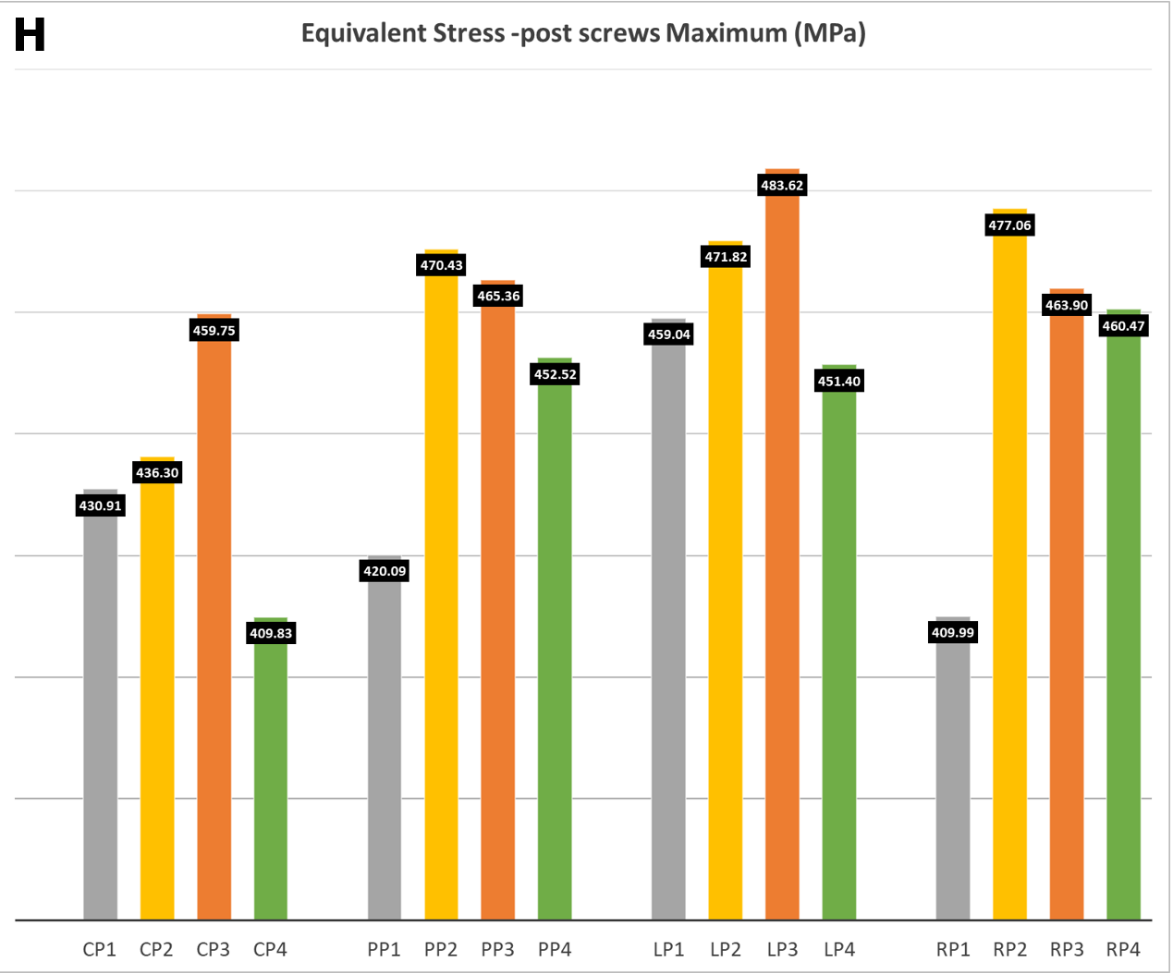


Figure S3H. Level of von Mises equivalent stress of posterior screws by FEA under the clenching (CP1-CP4), protrusion (PP1 to PP4), and left (LP1 to LP4) and right excursion (RP1 to RP4) conditions. conditions.


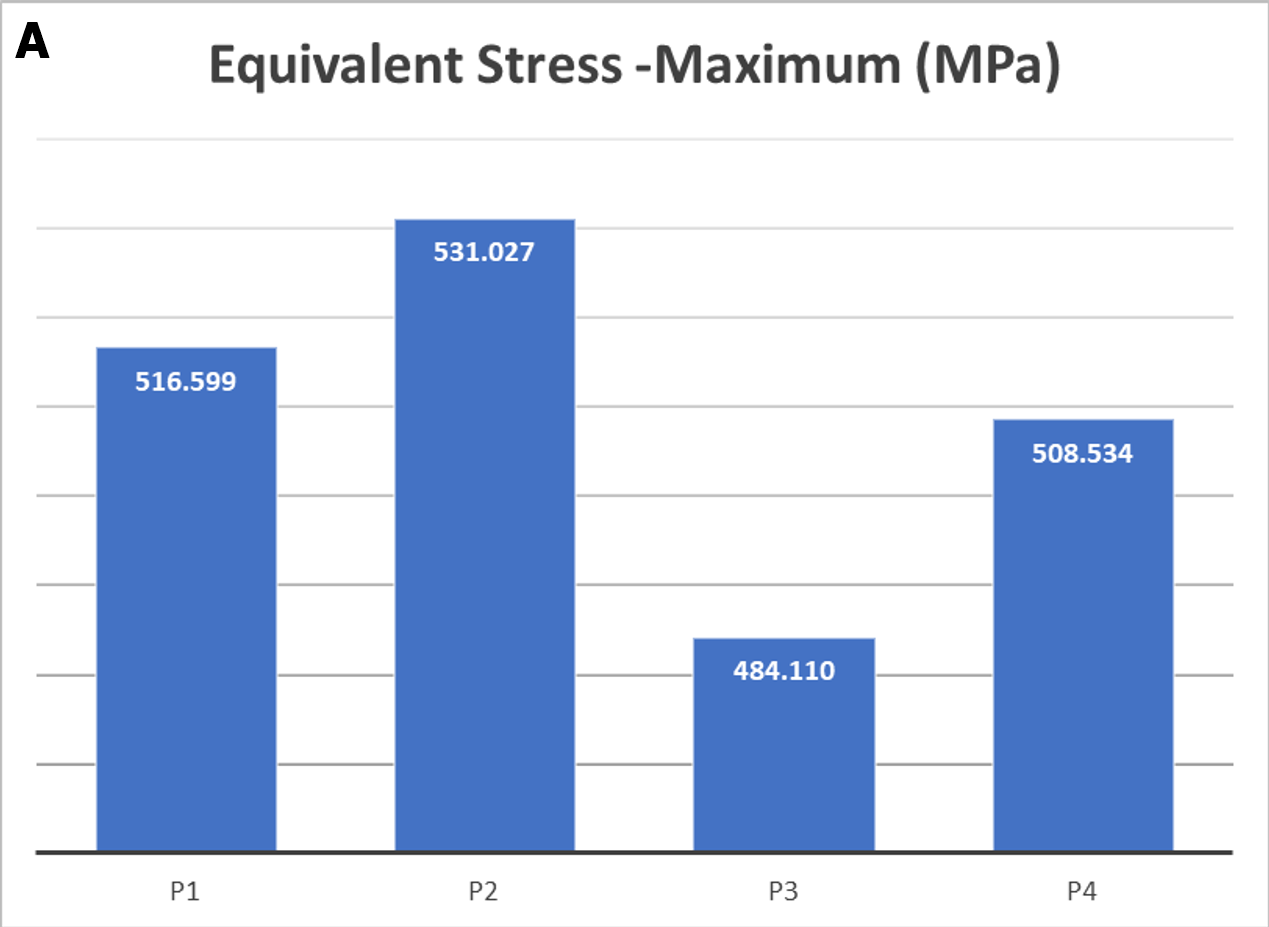


Figure S4A. Level of von Mises stress of whole components, CMP-PRD (P1, P2, P3, and P4) by FEA under the clenching condition in animal model.


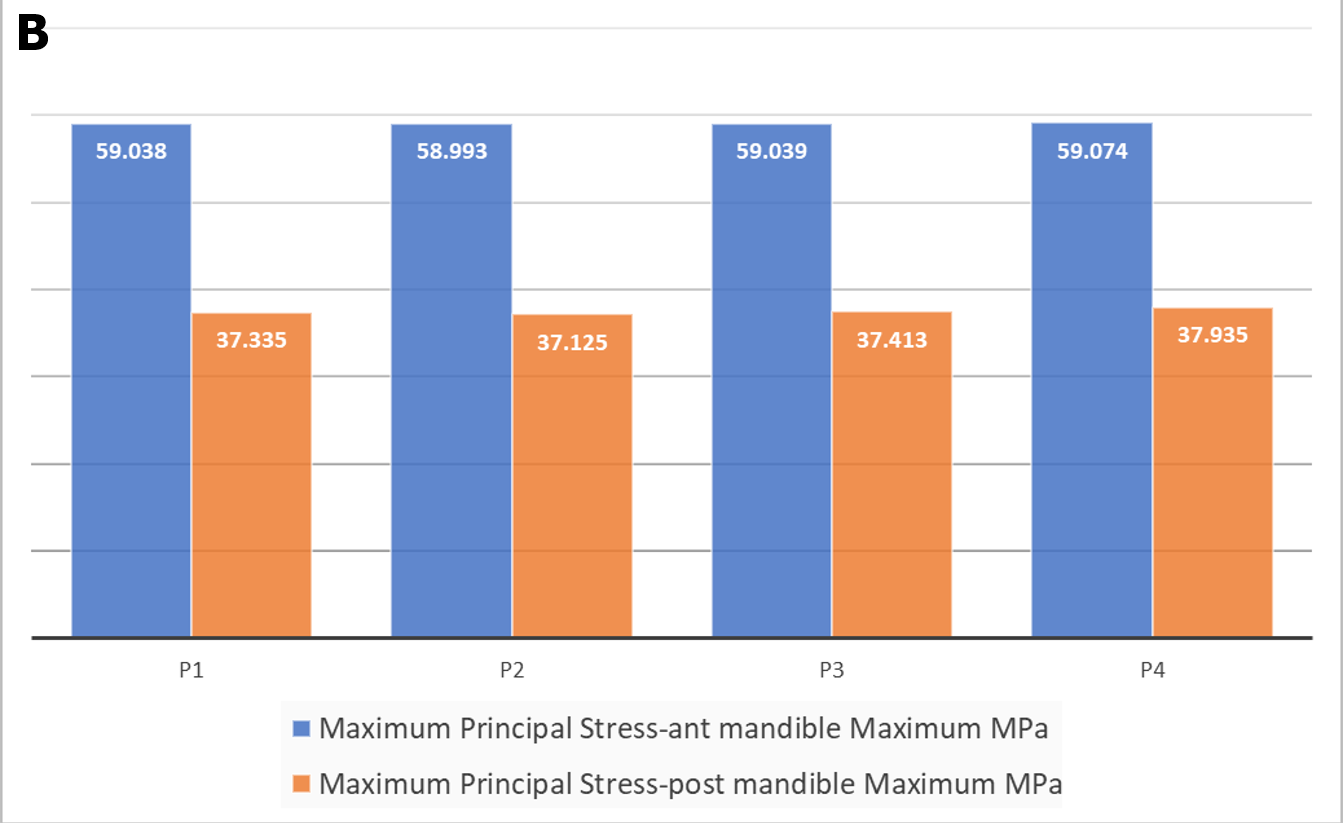


Figure S4B. Level of maximum principal stress of anterior and posterior mandible components of CMP-PRD (P1, P2, P3, and P4) by FEA under the clenching condition in animal model.


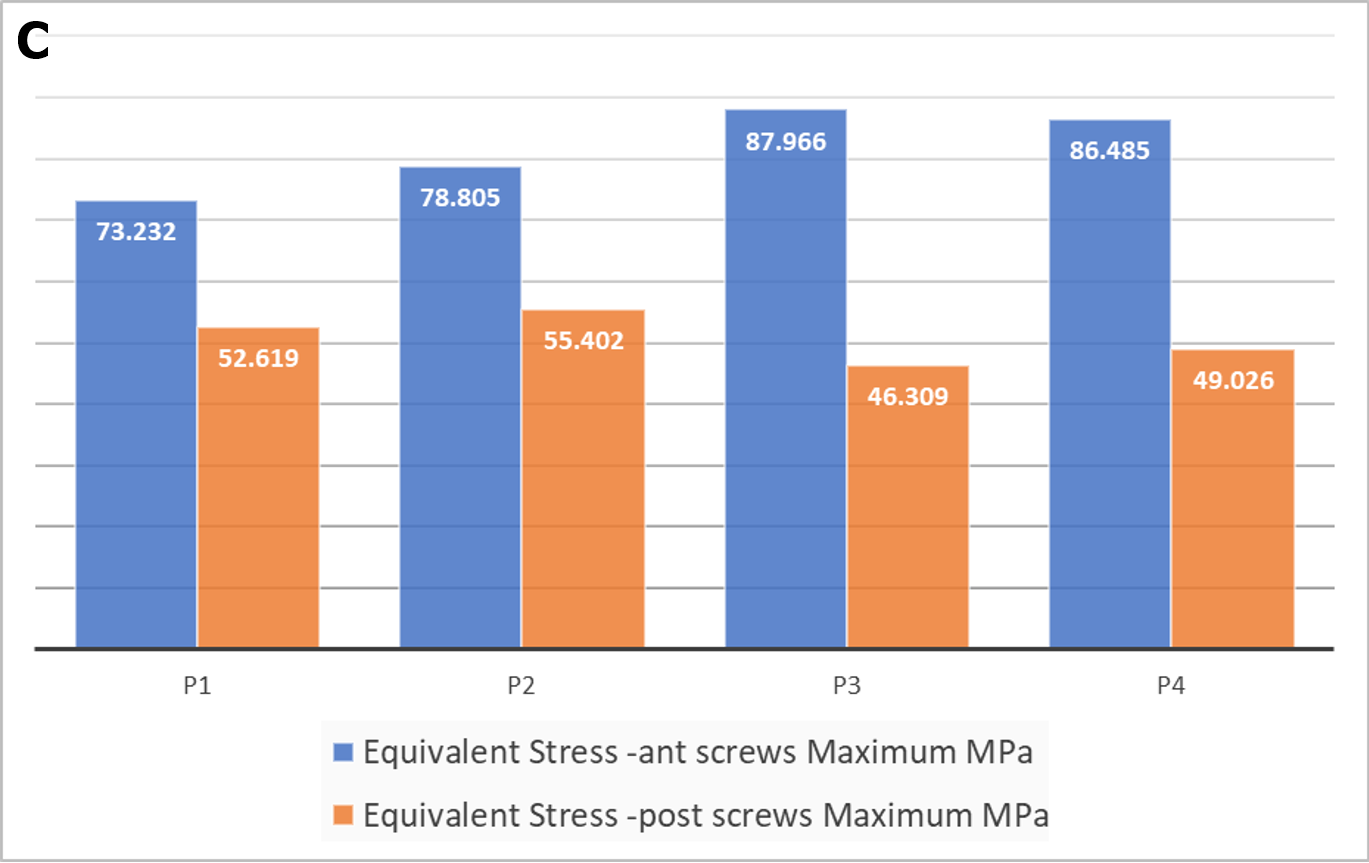


Figure S4C. Level of von Mises equivalent stress of anterior and posterior screws components of CMP-PRD (P1, P2, P3, and P4) by FEA under the clenching condition in animal model.


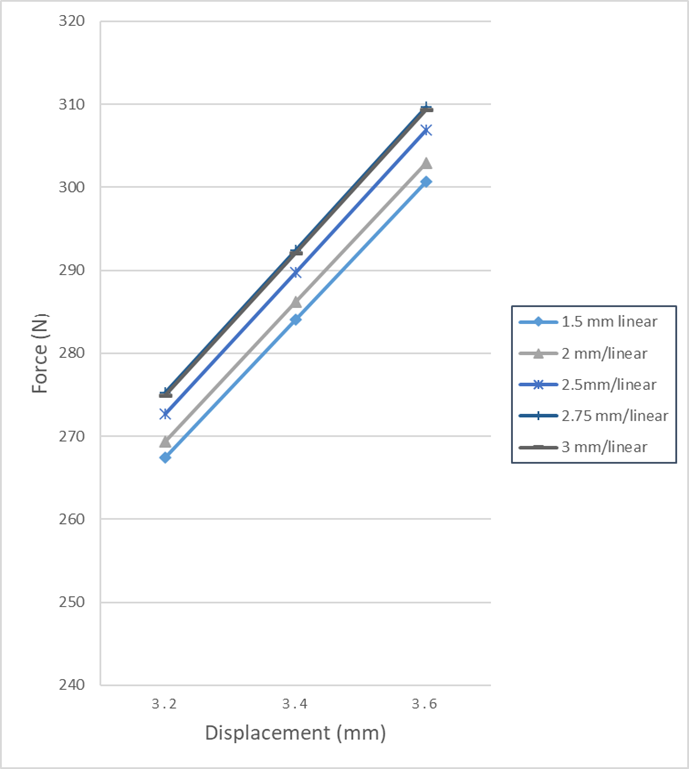


Figure S5A. Comparison of the force-displacement curves that were obtained from the FE models at different element sizes and linear formulations.


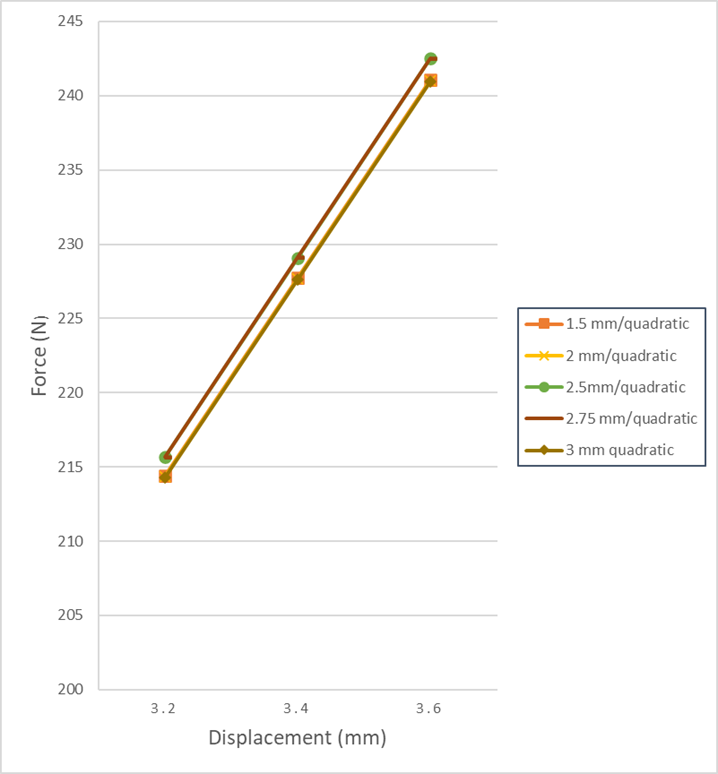


Figure S5B. Comparison of the force-displacement curves that were obtained from the FE models at different element sizes and quadratic formulations.
